# Dysbiotic gut microbes may contribute to hypertension by limiting vitamin D production

**DOI:** 10.1002/clc.23195

**Published:** 2019-05-28

**Authors:** Kun Zuo, Jing Li, Qiuhua Xu, Chaowei Hu, Yuanfeng Gao, Mulei Chen, Roumu Hu, Ye Liu, Hongjie Chi, Qing Yin, Yudan Cao, Pan Wang, Yanwen Qin, Xiaoyan Liu, Jiuchang Zhong, Jun Cai, Kuibao Li, Xinchun Yang

**Affiliations:** ^1^ Heart Center & Beijing Key Laboratory of Hypertension Beijing Chaoyang Hospital, Capital Medical University Beijing China; ^2^ Department of Endocrinology, Beijing Chaoyang Hospital Capital Medical University Beijing China; ^3^ The Key Laboratory of Upper Airway Dysfunction‐related Cardiovascular Diseases, Beijing An Zhen Hospital Capital Medical University, Beijing Institute of Heart, Lung and Blood Vessel Diseases Beijing China; ^4^ Medical Research Center Beijing Chaoyang Hospital, Capital Medical University Beijing China; ^5^ Hypertension Center, Fuwai Hospital, State Key Laboratory of Cardiovascular Disease of China, National Center for Cardiovascular Diseases of China Chinese Academy of Medical Sciences and Peking Union Medical College Beijing China

**Keywords:** gut microbiota, hypertension, vitamin D3

## Abstract

**Background:**

Accumulating studies have suggested that gut microbiota (GM) dysbiosis and vitamin D3 deficiency each play an important role during the progression of hypertension (HTN). However, few studies have characterized the underlying interaction between GM shift and vitamin D3 deficiency in HTN patients.

**Hypothesis:**

This study aimed to evaluate the possible crosstalk between GM dysbiosis and vitamin D deficiency in the pathogenesis of HTN.

**Methods:**

In a cohort of 34 HTN patients and 15 healthy controls, we analyzed the fecal microbiota products, GM composition, and the interaction between GM and vitamin D3.

**Results:**

Vitamin D3 was significantly decreased in feces of HTN patients (*P* = .006, vs controls) and was correlated with altered GM, including decreased Shannon index (*R* ^2^ = 0.1296, *P* = .0111) and Pielou evenness (*R* ^2^ = 0.1509, *P* = .0058). Moreover, vitamin D3 positively correlated with HTN‐reduced bacterial genera, including *Subdoligranulum* (*R* ^2^ = 0.181, *P* = .0023)*, Ruminiclostridium* (*R* ^2^ = 0.1217, *P* = .014)*, Intestinimonas* (*R* ^2^ = 0.2036, *P* = .0011)*, Pseudoflavonifractor* (*R* ^2^ = 0.1014, *P* = .0257)*, Paenibacillus* (*R* ^2^ = 0.089, *P* = .0373), and *Marvinbryantia* (*R* ^2^ = 0.08173, *P* = .0464). Partial least squares structural equation modeling showed that 27.7% of the total effect of gut microbiome on HTN was mediated by limiting vitamin D production. Finally, receiver operating characteristic curve analysis revealed the predictive capacity of differential gut microbiome signatures and decreased vitamin D3 to distinguish HTN patients (AUC = 0.749, *P* = .006).

**Conclusions:**

Our findings suggest that the GM dysbiosis contributing to the development of HTN might be partially mediated by vitamin D3 deficiency. Future studies involving the underlying mechanism and intervention strategies targeting microbiome composition and vitamin D3 to counteract the progression of HTN are warranted.

## 1 INTRODUCTION

Substantial progress has been made in understanding the etiology and pathophysiology of hypertension (HTN), which is a major risk factor for a variety of cardiovascular diseases and has become a global public health concern.^1^ HTN is believed to arise from the interplay between various environmental factors, and emerging evidence has demonstrated the causal relationship between gut microbiota (GM) dysbiosis and HTN.^2^ For example, aberrant GM composition was found in HTN patients and elevated blood pressure (BP) was observed after fecal transplantation from HTN human donors to germ‐free (GF) mice.^3^ GM may participate in the progression of HTN by modulating immuno‐inflammatory responses and regulating bioactive metabolites.^3^ A number of studies have confirmed the regulatory role of short chain fatty acids (SCFAs) in BP,^4^, ^5^ yet evidence to define the specific role of GM and its metabolites in the development of HTN is lacking.

In addition to GM dysbiosis, a large body of evidence has identified vitamin D deficiency as one of the novel risk factors for HTN. 6, 7 Underlying mechanisms for the progression of vitamin D deficiency‐related HTN may include increased renin expression,^8^, ^9^ hypocalcemia and hyperparathyroidism,^10^, ^11^ increased sympathetic nervous activity,^12^ and altered vascular function.^13^, ^14^ Importantly, crosstalk between GM and vitamin D was recently revealed by Cantorna et al,^15^ who showed the regulation of vitamin D metabolism by GM via fibroblast growth factor (FGF) 23. GM‐induced inflammation in GF mice inhibited FGF23 which led to increased vitamin D levels. However, studies of GM and vitamin D in HTN have been few in number and limited to animal models. Information regarding the interaction between GM and vitamin D in HTN patients is also incomplete. In order to evaluate the possible crosstalk between GM dysbiosis and vitamin D deficiency in the pathogenesis of HTN, we analyzed GM composition, performed metabolomic analyses of fecal microbiota products, and delineated the interaction of fecal vitamin D and GM in HTN patients compared with healthy controls.

## 2 METHODS

### 2.1 Study cohort

Thirty‐four HTN patients and 15 healthy individuals as matched controls (CTR) were enrolled from the Kailuan cohort.^16^ The cutoff BP for HTN diagnosis was >140 mm Hg for systolic BP (SBP), and >90 mm Hg for diastolic BP (DBP).^1^ Individuals with a history of heart failure, coronary heart disease, arrhythmia, structural heart disease, comorbidities, (inflammatory bowel diseases, irritable bowel syndrome, autoimmune diseases, liver diseases, renal diseases, or cancer) or use of antibiotics or probiotics in the previous month were excluded. Demographic and clinical characteristics were obtained by completing face‐to‐face surveys and checking hospital or medical examination records. The study conforms well to the principles from the Declaration of Helsinki. The research protocol was approved by the ethics committee of Kailuan General Hospital. All participants signed informed consents.

### 2.2 Metabolomic analysis based on liquid chromatography/mass spectrometry

Fecal metabolic profiles were obtained via liquid chromatography‐mass spectrometry (LC/MS). Detailed descriptions of LC/MS parameters are provided in the supplementary methods.

### 2.3 Analyses of GM composition

Metagenomic sequencing data of stool samples from our previous study^3^ were used in the present study, including abundance of 20 genera that distinguish HTN. Annotation, calculation of relative abundance, and correction of *P* values were performed as previously. 3 Furthermore, we calculated four parameters of GM composition including ratio of Firmicutes (F) and Bacteroidetes (B) (F/B ratio), Chao richness, Pielou evenness, and Shannon diversity. Detailed descriptions of these analyses are provided in the supplementary methods.

### 2.4 Statistical analysis

Quantitative demographic and clinical characteristic data with normal distributions were presented as mean ± SD and the *t* test was used for between‐group comparisons. Quantitative data with non‐normal distributions were presented as median (first quartile, third quartile) and the Wilcoxon rank‐sum test was performed for between‐group comparisons. Qualitative data were presented as a percentage and the *χ* ^2^ test was used for between‐group comparisons. All statistical tests were 2‐sided and *P* < .05 was regarded as significant. Statistical analyses were performed with SPSS version 22.0 (IBM Corp., Armonk, New York).

Correlation analyses were performed using the psych package in R version 3.3.3，and Mann‐Whitney test in GraphPad Prism 7 (GraphPad Software, La Jolla, California). Partial least squares structural equation modeling (PLS‐SEM) was conducted using the Smart‐PLS 3 software. Partial least squares‐discriminant analysis (PLS‐DA) was carried out using the SIMCA‐P software to cluster sample plots across groups.

## 3 RESULTS

### 3.1 Baseline characteristics of the study cohort

The clinical characteristics of 34 HTN patients and 15 CTR are shown in Table 1. Other than a significantly different SBP and DBP, there was no statistical difference between HTN patients and CTR (including age, sex, body mass index, total cholesterol, fasting blood glucose, creatinine, total bilirubin, and glutamic‐pyruvic transaminase). Of note, none of the participants were under antihypertensive treatment using medications such as angiotensin‐converting enzyme inhibitors, angiotensin receptor blockers, β receptor blockers, statins, or aspirin, which might influence GM.^17^

**Table 1:** Baseline clinical characteristics of the study cohort

	HTN Group	Control Group	*P* value
Number	34	15	/
Age, years	54.5 (49.25, 57.75)	58 (52, 60)	.102
Male/Female	31/3	11/4	.103
SBP	151(141, 160)	120 (118, 123)	<.001
DBP	95.5 (90, 102)	78 (71.67, 80)	<.001
BMI	25.56 (22.50, 27.97)	25.64 (24.32, 27.75)	.551
TC	5.07 (4.51, 6.00)	5.39 (4.87, 5.88)	.650
FBG	5.60 (5.22, 6.42)	5.23 (4.58, 5.83)	.107
Creatinine	65.5 (57.10, 84.5)	69 (64, 78.25)	.588
TBil	14.65 (11.08, 19.73)	17.54 (10.18, 23.10)	.413
ALT	21.1 (14.75, 27.25)	19.25 (12.75, 27.75)	.545

*Note*: Data are presented as mean ± SD, or median (IQR), as appropriate.

Abbreviations: ALT, glutamic‐pyruvic transaminase; BMI, body mass index; HTN, hypertension; IQR, interquartile range; DBP, diastolic blood pressure; FBG, fasting blood glucose; SBP, systolic blood pressure; TBil, total bilirubin; TC, total cholesterol.

### 3.2 Decreased vitamin D in feces of HTN patients

**Figure 1:**
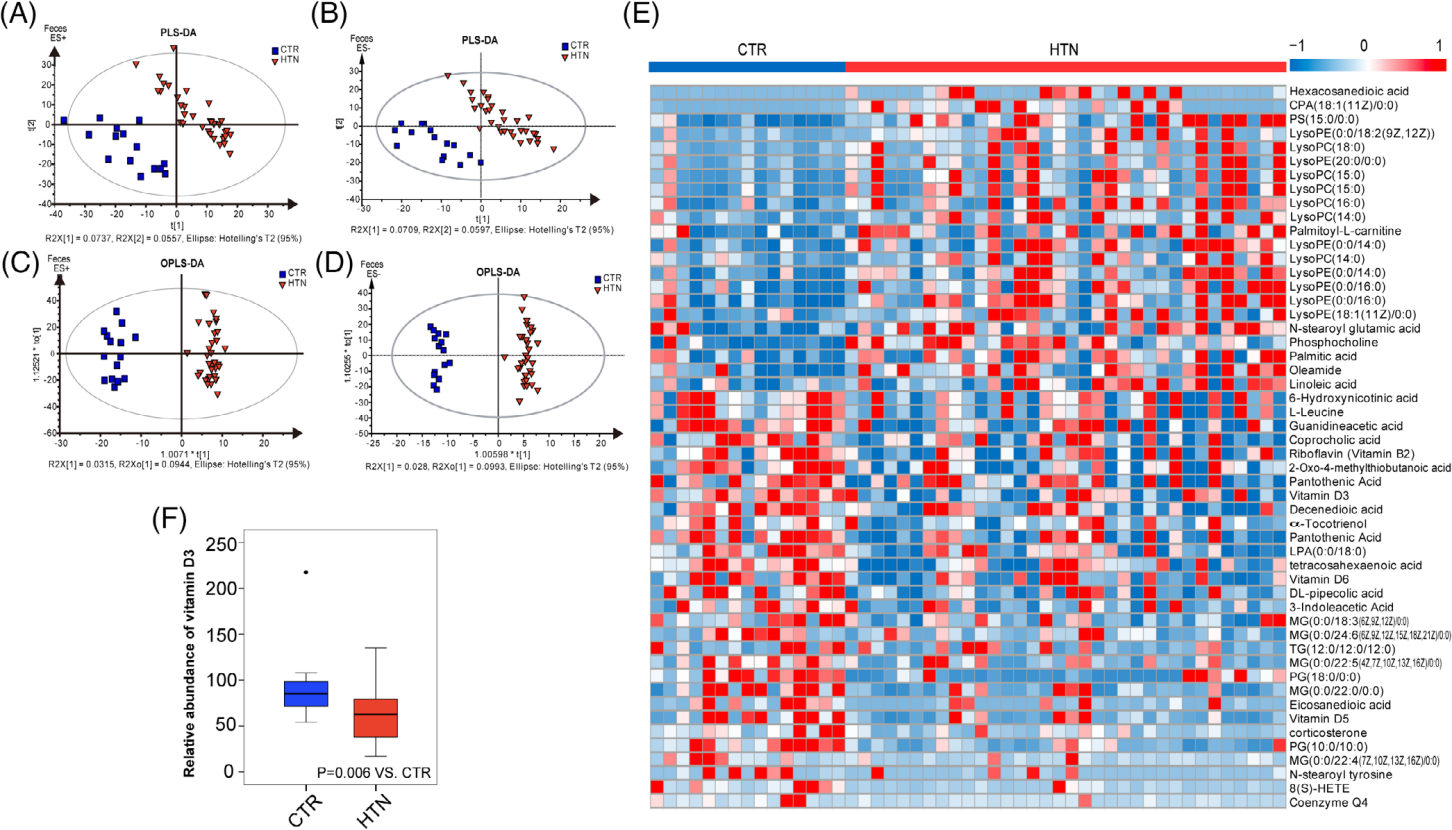
Decreased vitamin D in feces of hypertension (HTN) patients identified by liquid chromatography/mass spectrometry. A and B, Partial least squares‐discriminant analysis (PLS‐DA) score plots based on the metabolic profiles in feces samples from CTR and HTN in ES+ and ES−. C and D, Score scatter plots of orthogonal PLS‐DA (OPLS‐SA) comparing the feces metabolic differences to identify the separation between CTR and HTN in ES+ and ES−. E, Heat map showing the abundance of 52 endogenous compounds that varied in HTN feces transformed into Z scores. Metabolites significantly changed in HTN compared to CTR at VIP > 1.0 and *P* value (*t* test) <.05 are identified. F, Vitamin D3 was significantly decreased in HTN (*P* = .006, Kruskal‐Wallis test). Boxes represent the inter quartile ranges, the inside line or points represent the median, and circles are outliers

In order to investigate whether HTN is associated with a disordered intestinal microflora community, we performed high‐throughput LC/MS to explore GM metabolites in feces. PLS‐DA and orthogonal partial least‐squares discriminant analysis (OPLS‐DA) were plotted to reveal global metabolic changes between HTN and CTR. A clear separation between HTN patients and healthy controls was obtained under ES+ and ES− modes (Figure 1A‐D). Overall, 52 feces metabolites were altered (22 elevated and 30 decreased) in HTN patients (Figure 1E, Table S1). Of note, we found vitamin D3 was significantly decreased in HTN (91.29 vs 63.75, *P* = .006, Mann‐Whitney test, Figure 1F).

### 3.3 Disordered gut microbiome in HTN patients

**Figure 2:**
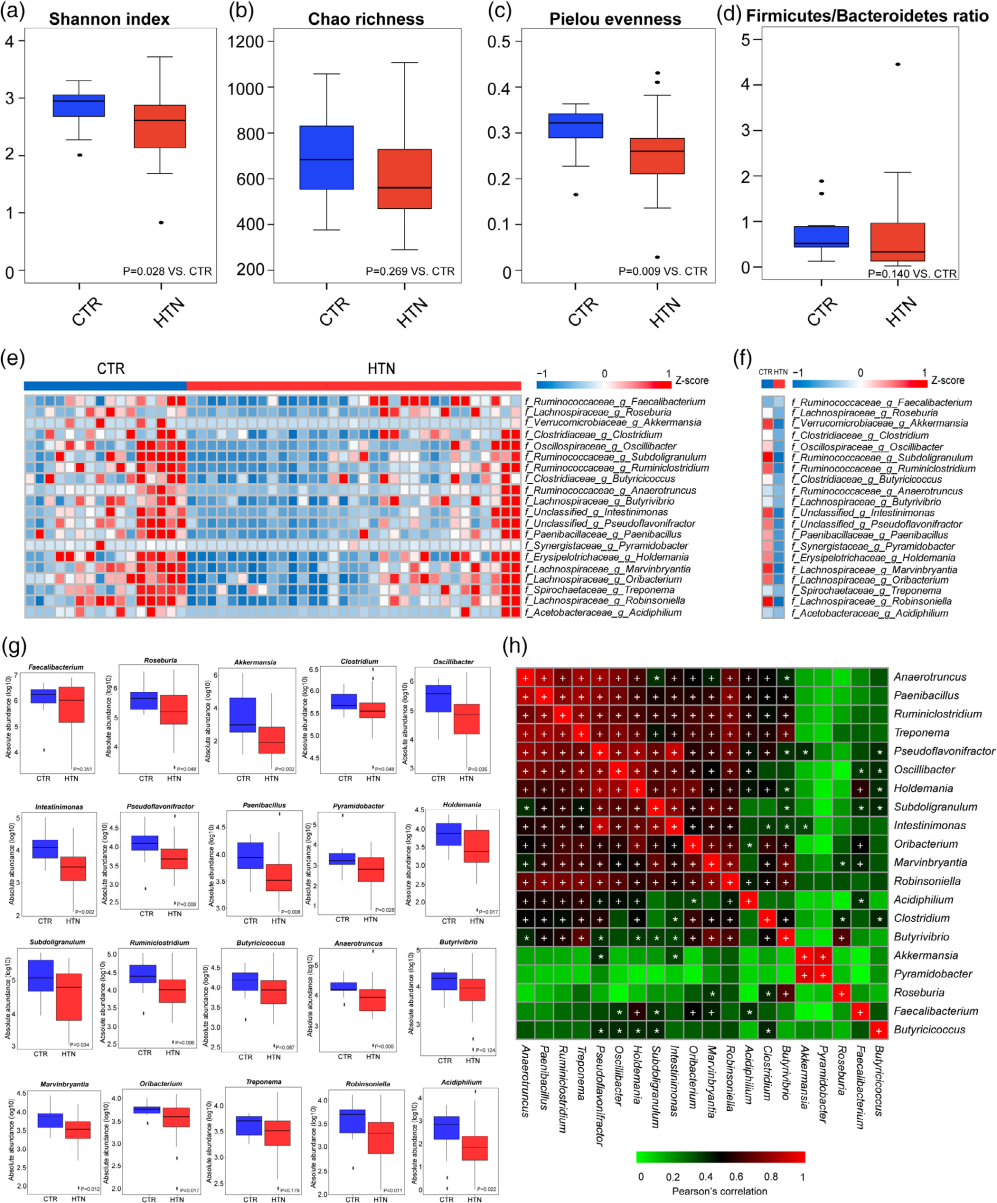
Disordered gut microbiota in the hypertension (HTN) gut. A‐D, Shannon index, Chao richness, Pielou evenness, and F/B ratio based on the genus profile in the CTR and HTN cohort (*P* = .028 for Shannon index; *P* = .269 for Chao richness; *P* = .009 for Pielou evenness; *P* = .140 for F/B ratio, respectively; Kruskal‐Wallis test). Boxes represent the inter quartile ranges, the inside line or points represent the median, and circles are outliers. E, Heatmap of HTN‐specific genera in 49 participants. The abundance profiles were transformed into *Z* scores by subtracting the average abundance and dividing the SD of all samples. *Z* score is negative (shown in blue) when the row abundance is lower than the mean. F, Heatmap of HTN‐specific genera in CTR and HTN. G, Boxplots of abundance of 20 HTN‐specific genera between CTRs and HTNs. Boxes represent the inter quartile ranges, the inside line or points represent the median, and circles are outliers. *P* value shown in the bottom of the plot, Mann‐Whitney test. H, Correlations of 20 HTN‐specific genera by using Pearson correlation analysis; Asterisks denote statistical significance between bacterial taxa, *P < .05, +*P* < .01

To evaluate the GM composition between HTN and CTR, we compared the F/B ratio and three other ecological parameters, including Shannon index, Chao richness, and Pielou evenness. Shannon index and Pielou evenness were significantly lower in HTN (*P* = .028 for Shannon index, Figure 2A; *P* = .009 for Pielou evenness, Figure 2C). Consistent with these measures, Chao richness and F/B ratio also trended lower in HTN, although not statistically significant (*P* = .269 for Chao richness, Figure 2B; *P* = .14 for F/B ratio, Figure 2D). The decreased richness of genera observed in our cohort may suggest an imbalance of harmful and beneficial bacteria in HTN patients.

From our previous study, 3 we identified 20 bacterial genera that discriminated HTN from controls, including *Roseburia*, *Akkermansia*, *Clostridium*, *Oscillibacter*, *Subdoligranulum*, *Ruminiclostridium*, *Intestinimonas* etc (Figure 2E‐G). The abundance of these genera is provided in Supplementary Table S2. Most of the HTN‐specific bacterial genera are considered to be beneficial microbes due to anti‐inflammatory properties or butyric acid production and were lower in HTN while enriched in CTR.^18^ Additional analysis of Pearson correlation indicated a positive inter‐group correlation among HTN‐specific genera (Figure 2H). Most of the texa decreased in HTN positively correlated with each other, suggesting that the imbalanced GM composition emerges as an assemblage during the progression of HTN.

### 3.4 Correlation of altered GM with vitamin D in the HTN gut

**Figure 3:**
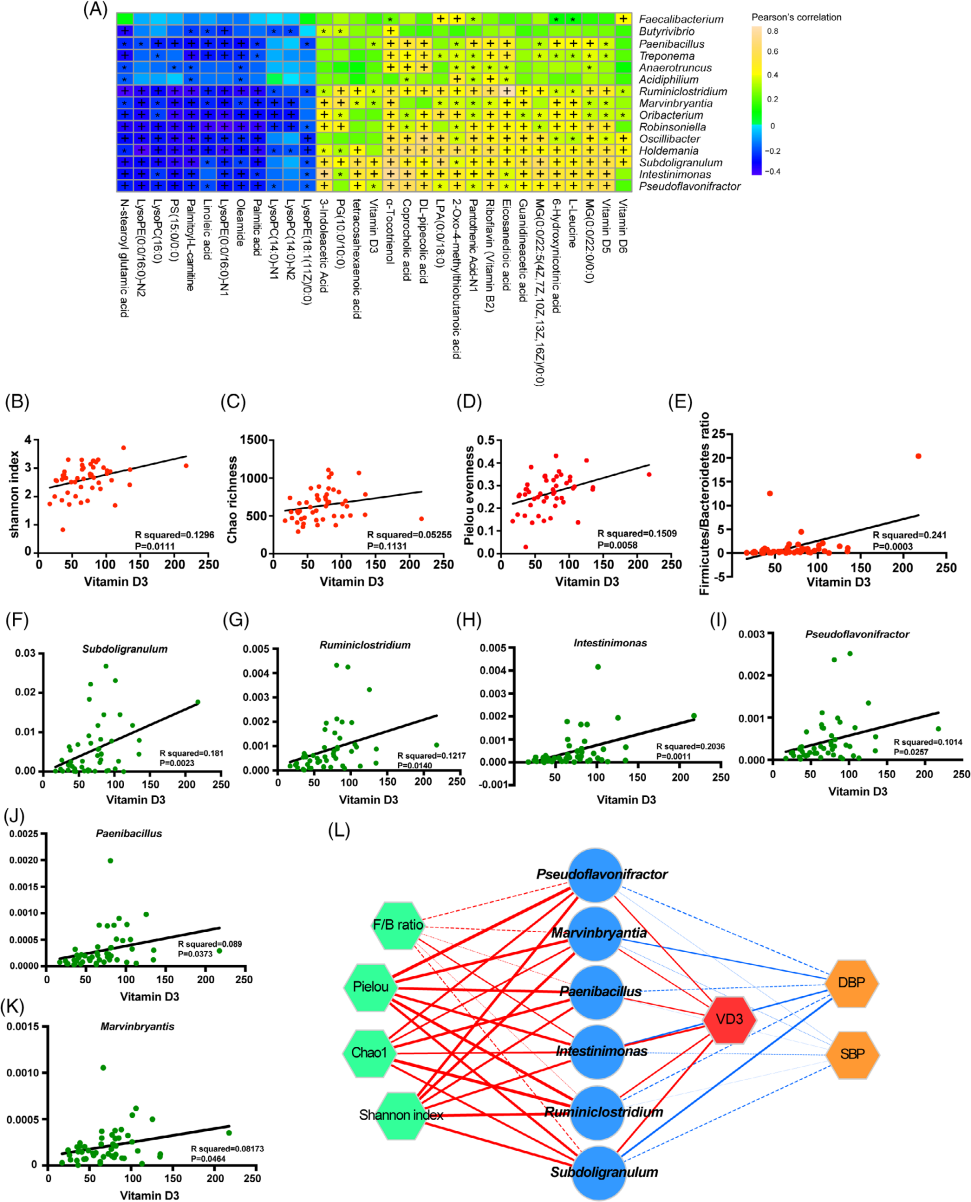
Correlation between vitamin D and hypertension (HTN)‐specific genera. A, Pearson correlation between the 20 HTN‐specific genera and 52 differential metabolites. Heatmap showed positively or negatively correlation between HTN‐specific genera and differential metabolites, where described the metabolites that were correlated with at least one genus with *P* < .05. Blue, negative correlation; yellow, positive correlation, *P < .05, +*P* < .01. F‐K, Correlation between vitamin D3 and six HTN‐specific generas (*R* ^2^ = 0.181, *P* = .0023 for *Subdoligranulum*; *R* ^2^ = 0.1217, *P* = .014 for *Ruminiclostridium*; *R* ^2^ = 0.2036, *P* = .0011 for *Intestinimonas*; *R* ^2^ = 0.1014, *P* = .0257 for *Pseudoflavonifractor*; *R* ^2^ = 0.089, *P* = .0373 for *Paenibacillus*; *R* ^2^ = 0.08173, *P* = .0464 for *Marvinbryantia*; Pearson linear correlations). L, Correlation network among six vitamin D3‐correlated HTN‐specific genera (*Subdoligranulum, Ruminiclostridium, Intestinimonas, Pseudoflavonifractor, Paenibacillus*, and *Marvinbryantia*), four gut microbiota (GM) parameters (Shannon index, Chao richness, Pielou evenness, and F/B ratio), vitamin D3, and systolic blood pressure/diastolic blood pressure (SBP/DBP) were performed by Cytoscape software. The nodes of the network represent the genera (blue circle), GM diversity parameters (green octagon), SBP (orange octagon) DBP (orange octagon), and vitamin D3 (red octagon) where the solid line correspond to a significant (*P* < .05) and the dotted line refers to *P* > .05. The red line means positive correlation and the negative correlation in blue line, where the line width indicates the Pearson correlations

Both decreased vitamin D3 and disordered GM were identified in HTN patients, which allowed us to speculate that crosstalk existed among GM and vitamin D3 in HTN. To explore the relationship between decreased vitamin D3 and disordered GM, several correlation analyses were performed stepwise. Firstly, a Pearson correlation analysis between HTN‐specific genera and differential metabolites was performed (Figure 3). Vitamin D3, which was remarkably decreased in HTN (Figure 3A), was correlated with HTN‐decreased genera such as *Paenibacillus*, *Ruminiclostridium*, *Pseudoflavonifractor*, and *Marvinbryantis*, which suggested an association between vitamin D3 and GM*. Ruminiclostridium* presented a positive correlation with 3‐indoleacetic acid and 6‐hydroxynicotinic acid, while *Pseudoflavonifractor* showed a negative correlation with linoleic acid, oleamide, and palmitic acid.

Then we analyzed the correlation between vitamin D3 and GM parameters. The positive correlation between vitamin D3 and GM parameters was striking (*R* ^2^ = 0.1296, *P* = .0111 for Shannon index, Figure 3B; *R* ^2^ = 0.1509, *P* = .0058 for Pielou evenness, Figure 3D; *R* ^2^ = 0.241, *P* = .0003 for F/B ratio, Figure 3E). The association between vitamin D3 and Chao richness had the same trend but without statistical significance (*R* ^2^ = 0.241, *P* = .1131, Figure 3C). These findings suggest that decreased vitamin D3 was partially associated with GM dysbiosis.

Moreover, to further clarify the role of specific bacteria with limiting production of vitamin D3, correlation analyses of 20 HTN‐specific genera and vitamin D3 was performed. The results showed that six of these genera were positively correlated with vitamin D3, including *Subdoligranulum* (*R* ^2^ = 0.181, *P* = .0023, Figure 3F), *Ruminiclostridium* (*R* ^2^ = 0.1217, *P* = .014, Figure 3G), *Intestinimonas* (*R* ^2^ = 0.2036, *P* = .0011, Figure 3H), *Pseudoflavonifractor* (*R* ^2^ = 0.1014, *P* = .0257, Figure 3I), *Paenibacillus* (*R* ^2^ = 0.089, *P* = .0373, Figure 3J), and *Marvinbryantia* (*R* ^2^ = 0.08173, *P* = .0464, Figure 3K). It is worth noting that *Subdoligranulum* is an anti‐inflammatory bacterium shown to be significantly depleted in patients with coronary artery disease.^19^ Furthermore, a previous study^20^ regarding inflammatory bowel disease (IBD), a condition in which vitamin D deficiency seems to play a role in intestinal inflammation, showed that specific gut species including *Subdoligranulum* were increased after oral vitamin D administration in IBD patients but not CTR. Moreover, as *Intestinimonas butyriciproducens* converts lysine into butyrate, which is an energy source for colonocytes, the ammonium released can be used as a nitrogen source by bacteria and neutralizes acidity created by SCFA production.^21^ The positive correlation between vitamin D3 and the reduction of specific GM genera in HTN patients suggests that GM dysbiosis may modulate vitamin D3 deficiency to mediate HTN.

To further identify the correlation among clinical indices, disordered GM and decreased vitamin D3, a correlation network (Figure 3L) containing these factors was performed. Both SBP and DBP were directly linked to dysbiotic GM and vitamin D3, which indicated that disordered GM was associated with decreased vitamin D3 and elevated blood pressure. Therefore, we may speculate that vitamin D3 production was limited by the disordered gut microbiota, which may contribute to the development of HTN.

### 3.5 Dysbiotic GM may contribute to HTN by limiting vitamin D production

**Figure 4:**
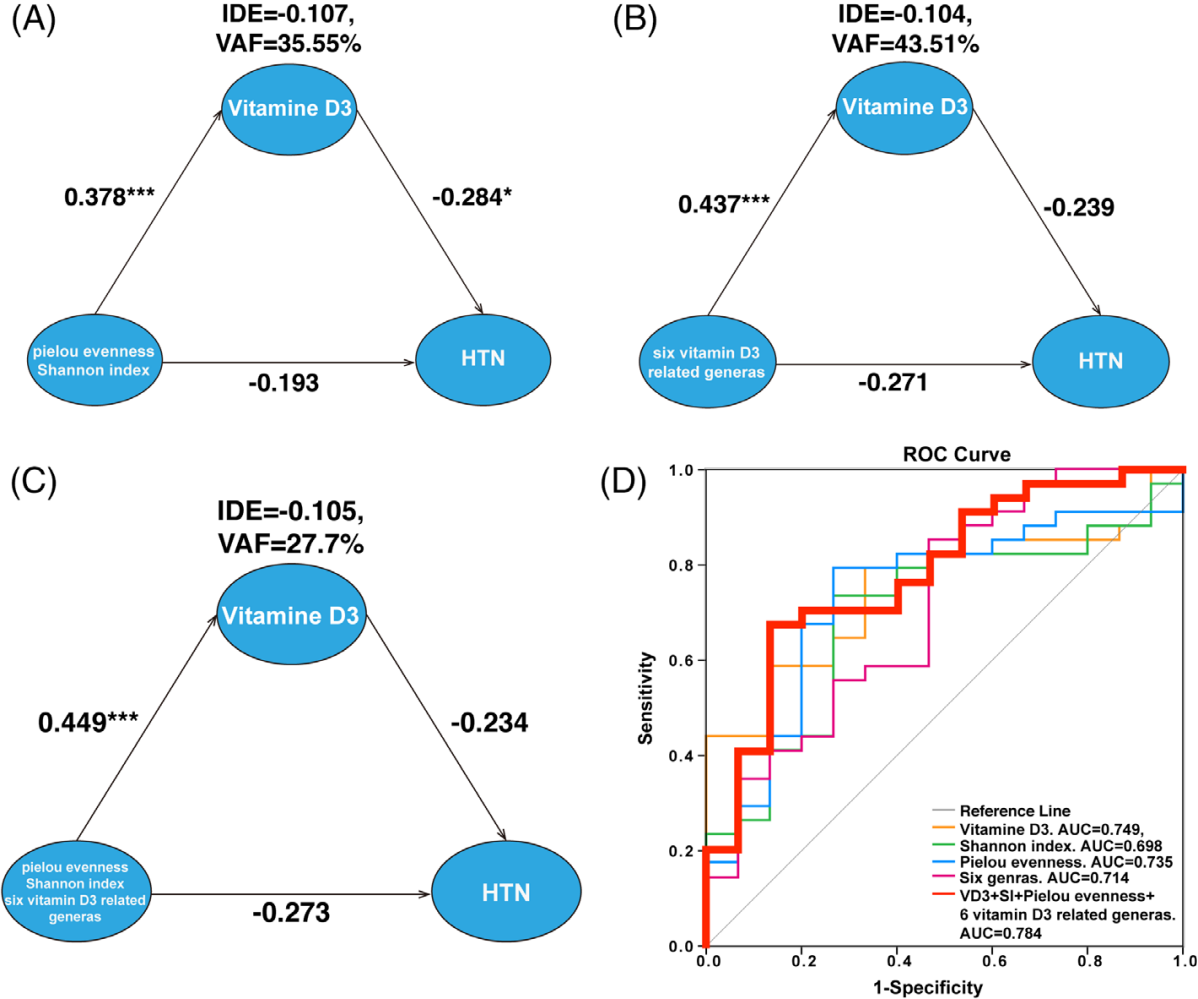
Dysbiotic gut microbiota (GM) may contribute to hypertension (HTN) by limiting vitamin D production. A‐C, Mediation analysis of the association between vitamin D3 and (A) Pielou evenness and Shannon index, (B) six vitamin D3‐related genera, and (C) all metrics combined using partial least squares structural equation modeling. Path coefficients are denoted beside each path and indirect effect and variance accounted for score are denoted below each mediator (*P < .05; **P < .01; ***P < .001). D, HTN is identifiable based on GM dysbiosis and vitamin D deficiency. Receiver operating curve for vitamin D3, Shannon index, Pielou evenness, and six HTN‐specific genera. The area under receiver operating curve (AUC) of vitamin D3 = 0.749 (95% confidence interval [CI]:0.613‐0.855, *P* = .006), Shannon index = 0.698 (95% CI: 0.542‐0.854, *P* = .028), Pielou evenness = 0.735 (95% CI: 0.583‐0.888, *P* = .009), six vitamin D3‐related HTN‐specific genera = 0.714 (95% CI: 0.613‐0.855, *P* = .006), and all metrics combined revealed an AUC of 0.784 (95% CI: 0.646‐0.923, *P* = .002)

Considering the correlation between vitamin D deficiency and GM dysbiosis, both of which contribute to HTN, we hypothesized that GM dysbiosis in the development of HTN was mediated by vitamin D3. Therefore, we applied PLS‐SEM to test if there was a mediating effect (indirect effect) of vitamin D3 during the GM shifts observed in HTN patients. We identified the variance accounted for (VAF) score, ratio of indirect‐to‐total effect that determines the proportion of the variance explained by the mediating process.^22^ The VAF for Pielou evenness and Shannon index was 35.55% (Figure 4A) while that of the six vitamin D3‐related genera was 43.51% (Figure 4B). The VAF for all of these metrics combined was 27.7% (Figure 4C). Thus, results from PLS‐SEM indicated that the contribution of GM dysbiosis to HTN was partially mediated by influencing the metabolism of vitamin D3.

### 3.6 HTN is identifiable based on GM dysbiosis and vitamin D deficiency

Based on decreased vitamin D3, dysbiotic GM and their crosstalk in HTN, we further implemented a receiver operating characteristic (ROC) curve to evaluate their diagnostic value. Analysis of ROC curves (Figure 4D) showed an area under the curve (AUC) of vitamin D3 = 0.749 (95% confidence interval [CI]: 0.613‐0.855, *P* = .006), Shannon index = 0.698 (95% CI: 0.542‐0.854, *P* = .028), Pielou evenness = 0.735 (95% CI: 0.583‐0.888, *P* = .009), six vitamin D3‐related HTN‐specific genera = 0.714 (95% CI: 0.613‐0.855, *P* = .006), and all of these combined revealed an AUC of 0.784 (95% CI: 0.646‐0.923, *P* = .002). In addition, the optimal cutoff value for predicting HTN by vitamin D3 = 80.898 (sensitivity 79.4%, specificity 66.7%) was set to verify the effect of vitamin D3 deficiency in development of HTN.

## 4 DISCUSSION

Mammalian metabolism is thought to be greatly influenced by an interaction with the intestinal microflora community. To explore how alterations in host metabolic patterns are impacted by the GM dysbiosis, data from metabolomics can supply certain information and indeed such reports have identified the contribution of metabolites from gut microbes to the development of several diseases. 3, 4, 23, 24, 25

In the present study, we obtained seminal evidence showing that the contribution of disordered GM in the development of HTN might be partially mediated by vitamin D3 deficiency. Based on LC/MS analysis, we found that vitamin D3 was significantly decreased in the feces of HTN patients and was positively correlated with GM composition parameters, including decreased Shannon index and Pielou evenness. Furthermore, vitamin D3 was positively correlated with HTN‐reduced genera, including *Subdoligranulum, Ruminiclostridium, Intestinimonas, Pseudoflavonifractor, Paenibacillus*, and *Marvinbryantia*. PLS‐SEM analysis showed that the contribution of GM dysbiosis on HTN was partially mediated by influencing the metabolism of vitamin D3.

These differential GM signatures and decreased vitamin D3 may be useful for the identification of HTN patients in clinical practice. Measurement of blood pressure is convenient, but BP can be highly variable. Thus, the detection and confirming diagnosis of HTN should be based on comprehensive evaluation, such as out‐of‐office or ambulatory BP monitoring.^1^ The relationship between BP and cardiovascular and renal events is continuous, making the distinction between normo‐tension and HTN based on cutoff BP values somewhat arbitrary.^26^ Therefore, based on our present results, detection of fecal vitamin D3 and GM composition could also be used as a biomarker during the diagnosis and follow‐up of HTN.

Emerging evidence suggests that GM disequilibrium can induce various diseases through disordered metabolic pattern and its influences on the targeted organ. For example, in the gut‐kidney axis disordered GM may lead to gut barrier dysfunction and bacterial translocation. Excessive uremic toxins are produced as a result of GM alteration, which are implicated in the kidney disease development. 27 Dysbiotic GM and metabolites can contribute to tubulointerstitial fibrosis, which might be mediated by tryptophan‐derived metabolite‐induced inflammation and oxidative stress.^28^ Meanwhile, the interaction between the gut epithelia and some commensal bacteria induces the rapid generation of reactive oxygen species, which participate in the development of nonalcoholic fatty liver disease.^29^

With the establishment of the heart‐gut axis concept, accumulating studies have suggested that the gut microbiome plays an important role in the genesis of cardiovascular diseases, including HTN. 3, 24 Our previous study depicted the signature of dysbiotic GM in HTN patients and demonstrated the causal relationship between GM dysbiosis and HTN.^3^ As the understanding of the relationship between intestinal microbiome and diseases has deepened, possible underlying mechanisms have been proposed. For example, emerging evidence suggests that immune and metabolic alterations might bridge GM dysbiosis and diseases.^4^ Butyric acid,^30^ one of the SCFAs produced as a GM metabolite, is a potent vasodilator. In fact, SCFAs have been recently shown to modulate blood pressure. Gut microbial products have been implicated in sympathetic activation and maintenance of an influx of lymphocytes to intestinal tissue.^31^ It follows that SCFAs from gut microbes contribute to the progression of HTN. Furthermore, there may be other GM metabolites that simultaneously participate in the regulation of BP, which warrants attention and further investigation.

Meanwhile, Vitamin D deficiency is an environmental risk factor that favors increased vascular tone. A significant body of evidence from animal models and observational human studies strongly supports the hypothesis that vitamin D deficiency contributes to HTN.^6^, ^7^, ^32^ The vitamin D receptor is expressed in many tissues including the cardiovascular system, located in vascular smooth muscle and endothelial cells.^13^, ^14^ Vitamin D regulates numerous genes involved in cell proliferation and differentiation, apoptosis, oxidative stress, membrane transport, matrix homeostasis, and cell adhesion.^33^ Vascular calcification is an active form of extracellular matrix mineralization by osteoblast‐like cells, mediated to a great extent by osteotropic hormones like parathyroid hormone and vitamin D.^34^, ^35^ Ectopic calcification in blood vessels is driven by stimuli like oxidative stress, phosphates, and oxidized lipids. The latter also induces macrophage‐derived signals, further promoting the mineralization process of calcifying vascular cells. 36

As mentioned above, HTN is caused by multiple factors acting through genetic and environmental determinants, and vitamin D deficiency and GM dysbiosis might act as environmental risk factors to favor increased blood pressure. A study^15^ bridging GM and vitamin D showed that GF mice have higher FGF23 and lower levels of vitamin D and calcium. FGF23 inhibits parathyroid hormone and induces cytochrome P450 family 24 subfamily A member 1 (Cyp24A1), which results in decreased vitamin D production.^37^ Disruptions in the microbiota and homeostasis of the gastrointestinal tract are also associated with IBD.^38^ Patients with active Crohn's disease have lower vitamin D levels.^39^ With more severe inflammation that occurs following *Citrobacter rodentium* infection at 2 weeks, vitamin D and 25D levels were lower. As the inflammation in the colon resolved, vitamin D levels increased in the *C. rodentium*‐infected GF mice. Disruption of the microbiota and inflammation had important and previously unappreciated effects on the availability of vitamin D to the host.

These interesting results are consistent with our present study from another aspect. Vitamin D3 was significantly decreased in feces of HTN patients and correlated well with GM composition parameters, such as Shannon index, Pielou evenness, and six HTN‐reduced genera. Additionally, we found the contribution of GM dysbiosis on HTN was partially mediated by influencing the metabolism of vitamin D3 using PLS‐SEM analysis. These novel findings provide preliminary clues for further studies exploring the potential crosstalk and mechanisms between gut microbes and vitamin D in HTN patients. Targeting GM to regulate vitamin D metabolism might provide additional benefit for HTN patients. An extensive amount of research is still needed to explore the clinical values of intervention strategies based on GM to improve HTN conditions.

Interestingly, besides the microbes in gut, some commensal oral bacteria have been revealed the role to shape the development of pulmonary HTN through the formation of nitrite, nitric oxide, which might contribute to the pathogenesis of obesity, HTN and cardiovascular diseases are linked to defects in nitric oxide signaling.^25^ Therefore, future studies linking the oral and gut bacteria may provide more valuable information.

The present study had several limitations. The number of participants was relatively small. Meta‐analysis^40^ of studies based on metagenome and metabolomics in HTN patients might provide additional evidence to strengthen the current results. Furthermore, the conclusions drawn from our data were associations rather than causal relationships. Studies investigating the underlying mechanism of how GM may limit the production of vitamin D are necessary.

## 5 CONCLUSION

The present study characterized the disordered GM and its contribution to the development of HTN, which was at least partially mediated by vitamin D3 deficiency. These novel findings are fundamental for further studies to explore the precise mechanism of how GM dysbiosis influences vitamin D3 and eventually participates in the pathogenesis of HTN. Intervention strategies targeting microbiome composition and vitamin D3 to counteract the progression of HTN are highly warranted and an extensive amount of research is still needed to explore the clinical value of intervention strategies based on GM and vitamin D3.

## CONFLICT OF INTEREST

The authors declare no potential conflict of interests.

## AUTHOR CONTRIBUTIONS

Xinchun Yang, Kuibao Li, Jun Cai, Jing Li and Kun Zuo conceived the study, directed the project, designed the experiments, interpreted the results, and wrote the manuscript. Qiuhua Xu, Qing Yin, Xiaoyan Liu and Yudan Cao collected the feces samples from the subjects. Yuanfeng Gao, Mulei Chen, Roumu Hu, Ye Liu, Hongjie Chi, and Pan Wang recruited, diagnosed, and collected the clinical details from the subjects. Kun Zuo, Jing Li, Jun Cai and Kuibao Li analyzed the data. Xinchun Yang, Jing Li, Yanwen Qin, Chaowei Hu, Jun Cai, Kuibao Li and Jiuchang Zhong revised the manuscript. All authors read and approved the final manuscript.

## Supporting information


**Appendix S1.** Supplementary methods: Description of detailed parameters in liquid chromatography/mass spectrometry. Analyses about parameters of GM composition.

**Table S1.** Detailed information of 52 metabolites differentially enriched across groups.

**Table S2.** Absolute abundance of 20 genera discriminative in HTN.

## Data Availability

Relevant data was within the paper.
