# Supplementary material for: Dysbiotic gut microbes may contribute to hypertension by limiting vitamin D production
Source: Clin Cardiol. 2019 May 28;42(8):710–9. doi: 10.1002/clc.23195 (PMC6672427; doi:10.1002/clc.23195)
Supplement: Supplementary file 1 — Appendix S1. Supplementary methods: Description of detailed parameters in liquid chromatography/mass spectrometry. Analyses about parameters of GM composition. [file CLC-42-710-s001.docx]

**Supplementary methods**

***Inclusion and exclusion criteria of subject***

The cut-off BP for HTN diagnosis was > 140 mmHg for systolic BP (SBP), and > 90 mmHg for diastolic BP (DBP). Individuals with a history of heart failure, coronary heart disease, arrhythmia, structural heart disease, comorbidities (inflammatory bowel diseases, irritable bowel syndrome, autoimmune diseases, liver diseases, renal diseases or cancer) or use of antibiotics or probiotics in the last one month were excluded. Demographic and clinical characteristics were obtained by completing face-to-face surveys and checking hospital or medical examination records.

***Description of detailed parameters in LC/MS***

Fresh stool samples were collected from each participant, immediately frozen at -20 °C, transported on ice to the laboratory and then stored at -80 °C. Then the fecal metabolic profiles were performed on a liquid chromatography-mass spectrometry (LC/MS) platform (Thermo, Ultimate 3000LC, Orbitrap Elite) using a Hypergod C18 (100 × 4.6 mm 3 μm) column.

50 mg fecal samples were pipetted into centrifuge tubes (1.5 mL) in preparation for extraction. The protein was precipitated with 800μL of methanol and 10μL of internal standard (2.9 mg/mL, DL-o-Chlorophenylalanine) was added. The samples were ground at 65 KHz for 90 s and centrifuged at 12000 g for 15 min at 4 °C. 200μL of the supernatant was transferred into a vial for further analysis. The fecal metabolic profiles were performed on an LC/MS platform (Thermo, Ultimate 3000LC, Orbitrap Elite) using a Hypergod C18 (100×4.6mm 3μm) column. The chromatographic separation conditions were as follows: the column temperature, 40 °C; flow rate, 0.3 mL/min; mobile phase A, water +0.1% formic acid; mobile phase B, acetonitrile +0.1% formic acid; injection volume, 4 ml; automatic injector temperature, 4 °C.

The following conditions were used for the positive ion mode (ES+): heater temp, 300°C; sheath gas flow rate, 45arb; aux gas flow rate, 15arb; sweep gas flow rate, 1arb; spray voltage, 3.0KV; capillary temp, 350°C; S-lens RF level, 30%. The following conditions were used for negative ion mode (ES-): Heater temp, 300°C; sheath gas flow rate, 45arb; aux gas flow rate, 15arb; sweep gas flow rate, 1arb; spray voltage, 3.2KV; capillary temp, 350°C; S-lens RF level, 60%.

All metabolomic data were prepared for feature extraction and pre-processed with Compound Discoverer 2.0 software (Thermo). Data were then normalized and edited into a two-dimensional data matrix by excel 2010 software, using Retention time (RT), Compound Molecular Weight (compMW), Observations (samples) and peak areas. Using SIMCA-P software (Umetrics AB, Umea, Sweden), a multivariate analysis was performed.

Compounds were significantly distinguished between groups, identified by a variable influence on projection (VIP) > 1 and p value < 0.05 based on the peak areas. The m/z value of these compounds was used to identify the metabolites related to the featured peak in the Metlin database. As for metabolites detected in both positive ion mode (ES+) and negative ion mode (ES-), the data in the mode with the higher VIP was retained for further analysis.

***Analyses about parameters of GM composition***

As increasing evidence indicated that changes in GM parameters could be potentially used as a biomarker for pathological conditions^6,29,30^, we calculated the ratio of Firmicutes (F) and Bacteroidetes (B), two main phyla that constitute the adult GM. Meanwhile, we evaluated the composition of GM communities based on 3 major ecological parameters^31^, including Chao richness (an estimate of a total number of operational taxonomic units present in the given community), Pielou evenness (to show how evenly the individuals in the community are distributed over different operational taxonomic units), and Shannon diversity (the combined parameter of richness and evenness). The Shannon index at the genus level was calculated with QIIME (Version 1.7.0), while Chao richness and Pielou evenness at genus level were calculated using the vegan package in R software (Version, 3.3.3).
