# Supplementary material for: Dysbiotic gut microbes may contribute to hypertension by limiting vitamin D production
Source: Clin Cardiol. 2019 May 28;42(8):710–9. doi: 10.1002/clc.23195 (PMC6672427; doi:10.1002/clc.23195)
Supplement: Supplementary file 2 — Table S1. Detailed information of 52 metabolites differentially enriched across groups. [file CLC-42-710-s002.pdf]

name	LysoPC(14:0)	Linoleic acid	LysoPC(15:0)	LysoPC(16:0)	LysoPC(18:0)	LysoPE(0:0/14:0)	LysoPE(0:0/16:0)	N-stearoyl glutamic acid	Oleamide	Palmitoyl-L-carnitine	Phosphocholine
CompMW	467.2986	280.2385	481.3153	495.3297	523.3602	425.2519	453.2826	413.3120	281.2700	399.3324	183.0650
CTR1	118.122135	398.805011	118.8404176	2490.581471	1429.22359	13.65354348	1025.133662	264.9374856	189.372	629.0949052	46.93554702
CTR2	75.28744655	616.469779	297.0564194	4383.241538	2710.787399	66.74400619	1487.461958	132.9387443	258.631	580.4274403	150.9952526
CTR3	38.19189466	342.12341	68.39174559	401.8636896	520.1220416	22.7901447	770.4429825	217.4785946	171.3807	1421.216475	69.05665648
CTR4	13.02935685	271.20137	54.69936622	380.7655472	233.6287517	9.914395693	286.8063361	36.99188457	161.7463	17.43852005	94.73918808
CTR5	78.54431307	538.629167	52.9830517	246.1047161	164.6142574	28.15652229	767.6343752	7.97065452	153.6298	14.66280916	55.59212506
CTR6	127.9460338	617.832336	50.74283374	678.8941063	418.8598882	40.97549654	1456.509468	21.13019183	350.2742	87.9706401	57.24900282
CTR7	83.26741318	289.956862	77.84220617	1310.538747	482.0556289	28.00886473	436.3052749	43.38898155	132.6869	162.5575264	40.47603724
CTR8	46.68192177	402.90244	166.8661346	3661.30824	1369.159443	14.00860069	1067.345886	59.48584271	181.4214	148.3455143	32.54376941
CTR9	17.19749999	202.81174	33.47935833	245.7229898	184.3200474	14.99253948	231.9504699	5.583520714	95.32748	12.28111657	54.11501689
CTR10	0.736494182	237.577054	67.28999632	1845.265612	709.7066119	2.406055069	292.7540243	61.82348339	105.1708	581.8528623	49.87225035
CTR11	73.19705459	367.82344	152.9472765	1349.499775	833.1088985	44.85616541	580.3191368	22.32266879	147.0957	23.81164369	55.55122479
CTR12	15.53083095	451.403919	39.05498034	194.4807361	181.2018433	16.40509075	234.9445601	11.85823024	88.32362	54.46446421	55.2378641
CTR13	39.18187683	570.406916	28.27029815	143.6138586	142.1340308	18.83194417	346.5095281	1.729817345	112.7225	17.12974236	62.37556698
CTR14	22.81411265	355.886665	64.07769381	1460.651564	1133.801598	9.130098139	320.4177963	87.91798226	127.1413	152.1866967	50.20738353
CTR15	10.88125009	373.062321	43.88559275	1087.446847	720.8680223	10.79711798	287.2773517	80.5021133	110.5181	448.9211619	46.91383301
HTN1	16.70394268	444.022718	256.40093	2779.314773	1988.870196	49.89258945	598.1215353	24.24863596	162.5721	63.09120997	156.5355144
HTN2	81.51348659	712.516992	128.7729187	2317.522713	2286.660746	82.90372837	2141.759462	139.6275854	379.9504	1881.238156	102.6336986
HTN3	40.70435982	219.614045	871.959958	12002.9006	16990.58107	27.68477358	543.7907339	51.91529874	169.5171	1015.799965	253.8817337
HTN4	42.42827922	293.310136	53.71066195	401.9893872	518.6768072	55.58787112	790.2171501	104.1547243	219.3804	1000.2118	86.35549732
HTN5	68.29657998	488.606909	54.61646817	1039.102379	667.6940182	31.47350491	703.6052394	187.4538261	351.2557	1062.191135	81.87741895
HTN6	13.86124378	375.71144	65.68147195	1451.303798	470.4674853	7.288990737	527.0633643	51.99562657	180.9808	486.1146307	85.75128381
HTN7	91.08288029	478.794767	217.155554	3841.007137	2432.517584	27.0018306	1322.515088	296.8646725	180.5694	721.3473434	133.3580292
HTN8	152.01699	431.419666	219.1361274	3128.489456	1329.634039	35.85726849	941.7789448	94.50134297	190.3889	238.3722484	187.9809357
HTN9	179.0915495	454.143692	447.162953	1184.077938	1594.614921	12.92568379	1087.20546	243.5360732	205.6332	41.48265931	186.0899701
HTN10	26.68380124	276.175108	106.6358492	624.0802155	677.5767008	8.851985909	565.132805	201.9971193	287.3992	106.9976662	99.93314645
HTN11	61.3749724	376.293072	81.64997037	773.730399	1153.41078	39.73626622	938.951898	69.91444189	193.8592	850.7460731	71.38017787
HTN12	231.2910958	635.913757	633.3302806	8090.675565	6687.290691	72.17577901	1611.569793	123.487138	310.903	1782.654428	87.38876625
HTN13	2.786480746	203.876102	10.16234709	53.17936181	61.82105283	13.28010201	73.27580489	14.57424479	172.7017	111.9723344	132.2361965
HTN14	67.77719681	1043.70462	268.8829491	2588.284488	4356.969116	165.8678107	2330.254293	108.4392315	354.011	196.4348413	120.1339303
HTN15	468.6927333	946.964405	620.3514633	7645.001682	7783.950542	151.2248215	3131.786944	150.5103297	249.182	903.0610129	131.0150022

name	3-Indoleacetic Acid	2-Oxo-4-methylthiobutanoic acid	6-Hydroxynicotinic acid	8(S)-HETE	corticosterone	DL-pipecolic acid	Guanidineacetic acid	L-Leucine	LPA(0:0/18:0)	MG(0:0/22:0/0:0)	MG(0:0/22:4(7Z,10Z,13Z,16Z)/0:0)
CompMW	175.0623	148.0152	132.0971	320.2338	346.2103	129.0784	117.0572	131.0941	438.2746	414.3743	406.3059
CTR1	12.87518418	515.0557549	608.0386491	245.738985	11.05962347	86.92460979	140.1145755	8276.738518	4.967816156	1.220193973	1.265416346
CTR2	6.448252738	640.3504799	379.6064599	26.9050733	18.70885592	374.9084077	208.3196296	5281.045198	4.575717799	30.47859274	27.30390032
CTR3	79.20225599	1028.126728	685.657268	19.7279519	14.83097365	193.7898898	326.8762561	9422.702506	2.742497987	104.863117	9.096216468
CTR4	27.25808334	1145.287142	851.5000107	126.285487	10.03642371	102.6715597	414.6928696	11453.05341	4.402336229	37.57695642	216.3101711
CTR5	37.5466723	1295.972044	728.7287176	45.3952067	28.34537099	369.4401426	414.3750887	10058.41936	18.71713863	291.6878008	44.35618656
CTR6	16.62218329	1131.615895	490.9338216	53.0302049	24.22069812	410.9788949	335.434964	6702.340393	6.853238625	205.4414698	30.87725153
CTR7	31.59776464	1002.150595	543.4536737	28.984277	19.67227671	170.2464166	290.8515052	7463.735724	7.997596742	37.12343559	8.134332283
CTR8	16.57373206	383.5235172	505.6470204	9.31234141	2.544699078	80.68889645	161.6200315	6618.209389	4.34390315	232.7499567	7.121285562
CTR9	52.43433861	641.3980925	430.9226725	18.727362	6.737350614	337.5833379	282.4825697	5879.118357	2.829943565	516.2293347	6.853613412
CTR10	136.9990088	896.6806084	504.7864161	48.2225582	20.62944617	71.87960277	257.205351	7126.171184	10.81982793	14.31999418	0.134700059
CTR11	22.60624254	848.2697121	561.0575777	38.3469715	12.7613673	138.3017074	278.8384231	8025.580164	12.91927668	113.4730613	22.92227285
CTR12	35.97686664	1362.494708	566.8073466	936.517185	208.2025964	163.0145091	244.5903668	7777.082628	9.582118354	220.7045365	3.814535733
CTR13	113.1588044	2054.395576	1109.207188	353.175239	13.02446152	256.125937	344.0135791	14777.58482	6.556051547	363.1178227	0.448154306
CTR14	29.42978965	1762.784739	707.7382985	127.314697	127.5753661	639.6228163	363.6208943	9600.565307	7.123731331	62.66215708	4.180066554
CTR15	31.40736256	1227.866195	624.3188984	70.6512247	77.48551187	332.7659335	272.7252382	8626.184553	8.087107642	228.1168223	4.894526174
HTN1	54.900219	587.0370747	462.2638012	8.94046337	19.74105619	42.05820374	192.3470302	6641.528386	4.698545648	46.94265101	7.048139315
HTN2	6.89108145	911.6726851	430.8042005	17.908906	15.75213959	110.2734079	376.3459756	5994.505365	4.115670013	48.97680792	18.20178237
HTN3	13.76094857	210.1448027	823.101052	6.11697898	1.223997922	167.4035044	139.1173361	11234.30562	2.264936472	16.62369043	0.697170607
HTN4	13.6332058	734.7615799	434.0417035	13.7608601	1.685343276	129.3212305	158.6410777	5742.662675	0.529254483	1.060180918	2.369429874
HTN5	5.401539195	396.9480481	503.3810231	7.54405657	13.1357013	44.29475231	132.5068286	6615.653664	2.267340016	6.980537344	0.299908253
HTN6	7.765410681	806.3196869	372.4065985	1.64898209	1.757230028	23.94737136	120.171038	5194.709291	0.134491417	29.24830517	0.23599563
HTN7	68.74300613	1382.508032	437.1756826	10.3715057	8.397155216	95.1861868	166.2679966	6101.294425	7.573949232	10.42767143	6.34576651
HTN8	47.92526567	2078.273971	618.4096338	22.1210931	23.32922571	115.641333	285.6549252	8467.811358	8.433320811	40.86509797	0.753965634
HTN9	11.57232375	792.153416	548.7308578	11.3511378	5.968137045	184.8167723	229.5100722	7488.023939	4.210312908	193.0293374	2.538440589
HTN10	35.20109701	845.5573422	474.0578196	11.4908865	3.235359842	161.5464544	230.6476422	6408.473498	19.74920254	69.42589144	2.43501829
HTN11	5.562657625	370.6166981	384.5846082	35.5500076	46.38343367	62.23503736	267.2918377	5284.092703	8.887334563	143.3100452	4.941797471
HTN12	4.71658786	265.4526737	443.9209272	10.4734432	8.627550365	85.24641251	161.4418863	6220.188762	2.384473422	28.09731494	30.0959449
HTN13	16.42526909	367.246514	867.1249323	4.69789955	7.994119485	117.8344585	124.076417	11147.49661	2.981367135	22.11118528	0.351701385
HTN14	14.73964161	625.1946688	442.7383375	8.16774402	6.116131673	38.01194775	175.8739589	6235.679911	1.495285181	23.22518826	6.705052022
HTN15	3.918367687	370.0402896	348.906766	8.92215809	4.42635067	59.39718997	155.523931	4909.772659	5.825708182	45.3071204	1.595626973

HTN16	530.5280773	518.521034	184.5619333	290.4217659	105.8211917	108.1081256	1368.058138	80.68371682	182.7782	152.8887964	117.7810064	26.75541255	689.2798152	606.8200195	8.41710977	23.89921179	221.5897043	240.2116769	8240.323197	4.686186988	22.0505072	4.752668093
HTN17	66.99953524	542.987237	130.8628682	5078.467433	2397.830548	64.84234758	1253.204328	38.06746664	136.4921	528.7681007	193.8101988	15.50235179	936.5967117	541.2961641	267.108951	14.8567586	46.59372036	320.6588494	7590.835142	6.604206664	250.1444185	3.972183076
HTN18	51.1517532	327.450621	76.47478872	1637.021555	716.5943457	36.7681967	585.6907351	77.36539642	145.999	1126.707892	63.98900879	11.40057532	776.4649874	535.4781927	66.1122515	11.25095861	382.5597322	298.4743124	7133.990461	6.198444529	171.9144121	58.80199615
HTN19	17.3996911	216.389529	14.22554642	163.4193989	103.5953675	16.80573133	80.2968412	2.550400905	71.55033	17.67558611	104.4563176	54.81591176	532.4948202	672.6549617	41.8191972	30.03629016	329.5224515	359.990171	8728.537598	7.131874281	291.9529184	8.387117671
HTN20	326.7334208	806.616861	462.4544615	4228.967281	2466.238079	61.03279032	2130.574309	29.88557775	212.8393	149.0101737	162.400706	30.01208433	1111.825582	628.1147673	47.036327	20.04846258	271.6290986	449.7272999	8492.724197	4.251949725	3.540756412	2.218821097
HTN21	69.0940976	623.914024	485.332508	5813.42728	6650.327073	84.75117511	1361.989534	185.6535096	328.747	848.498271	96.76749606	13.11494755	332.7553141	218.0366344	9.8196976	7.215801923	67.8175522	131.8913294	3017.789628	6.857996866	5.863116265	0.156980903
HTN22	150.0996908	706.359263	331.4381766	2008.028244	1223.848889	51.1923987	1828.577636	74.97748231	261.4717	154.3790689	63.27757095	22.61933158	835.142984	458.2536876	22.8951333	27.77772503	192.1597835	411.7129502	6293.94029	1.584088468	74.68709964	6.191154715
HTN23	44.85564557	1242.02284	72.97129666	5821.77968	1470.984175	21.53214152	863.4600967	157.5266486	243.2084	2605.283281	47.81764387	3.501673322	305.6152343	535.4098315	3.17097369	1.966568778	34.0425002	111.8088674	7203.954587	0.594693248	76.76492049	2.152474267
HTN24	116.7180745	416.271206	250.0999506	2035.178444	663.757733	28.08955745	900.5877267	29.7059688	182.7237	29.62895309	65.12670051	58.49657837	886.8047533	709.4806358	27.4000689	16.86552567	365.0231565	404.5562559	9257.975555	9.163435417	27.75756439	4.649692059
HTN25	8.927295553	955.222151	184.0328766	4997.887165	3734.238761	23.79055327	946.5447555	210.1651468	144.408	843.6965684	99.77107648	6.657489318	805.5183287	370.0144664	16.5604431	6.257244712	83.39226112	133.9737399	5208.973219	1.077205996	39.89317618	1.995050012
HTN26	27.47030386	360.620124	42.57801845	912.8246503	449.5294442	10.54098667	558.9556605	157.9698266	151.4012	1439.642795	84.10891287	69.39187839	1722.977215	720.5216521	26.6977972	6.124266875	91.57152694	244.873037	9889.715008	5.957371045	39.70975674	6.485881522
HTN27	103.2744161	610.368833	100.0416032	1833.891736	743.4820695	88.93350665	1400.713567	129.1320912	255.7334	417.7390664	66.49286838	8.681447993	365.2241032	336.2254301	4.46898583	5.207892594	27.21830836	106.4696958	4795.625743	3.077353092	23.42848112	6.450788353
HTN28	224.4787596	1166.21603	407.8412126	8709.474673	6623.618853	82.46092484	3459.821123	375.8489186	416.5325	1848.484633	105.4098808	12.62908262	315.3633607	178.5232065	8.33132044	11.2350702	82.49499067	172.7453011	2516.926894	2.530929677	30.65194598	2.810252309
HTN29	40.84545017	257.713846	70.42783422	649.8912248	264.3180569	168.7767155	356.6899779	8.124553095	105.0521	19.22956783	72.90762542	20.91878223	1434.242102	679.0298841	9.83368641	17.81893482	526.1201562	260.3021626	9155.359071	5.79580521	135.6424596	2.753542345
HTN30	231.7445506	1262.3044	578.9679805	10045.47607	6179.34149	87.44542155	3918.853747	134.0113525	309.1573	260.1009489	102.5841004	10.02227953	907.3056728	260.2786521	13.8779794	6.263370817	108.9693377	148.7647136	3580.015175	0.199223467	0.696458537	0.864217202
HTN31	31.76771881	341.557444	844.6201191	4863.49585	4081.539563	60.76189016	600.4408922	234.4702495	185.7287	730.8102577	94.92044035	33.03127674	814.9784593	856.9596278	5.03613528	5.934374321	126.4640608	123.9764305	12011.58227	2.172462091	9.895548889	3.67515383
HTN32	124.2305721	753.691655	70.77172311	870.7388612	392.9686505	51.16583344	2326.393689	291.8613992	360.641	1643.914932	52.47592527	6.381402373	343.1519596	451.3776252	25.5634783	4.741373767	60.39462666	391.0646392	6202.218581	0.353497269	0.78161033	10.92701763
HTN33	400.1261877	727.344398	177.5995728	3797.126698	1180.840868	76.43770172	2048.074197	127.8110277	257.3311	512.321551	62.00572698	20.07640287	724.8867956	629.2833391	13.2844712	7.266494377	54.24389892	231.2626341	8651.764063	1.910307012	25.4737609	7.943813474
HTN34	84.11759851	665.216592	770.5087884	3759.381703	6532.748317	46.00021185	2052.304939	120.7523722	532.0122	1384.142439	83.23343082	16.21318557	755.9544074	391.8736088	8.725268	9.74653533	126.9285083	145.38396	5517.013517	1.599439001	52.83597465	0.79897488

MG(0:0/24:6(6Z,9Z,12Z,15Z,18Z,21Z)/0:0)	N-stearoyl tyrosine	Pantothenic Acid	Riboflavin (Vitamin B2)	tetracosahexaenoic acid	Vitamin D3	Vitamin D5	Vitamin D6	α-Tocotrienol	LysoPC(14:0)	Coenzyme Q4	Coprocholic acid	CPA(18:1(11Z)/0:0)	Decenedioic acid	Eicosanedioic acid	Hexacosanedioic acid	LysoPC(15:0)	LysoPE(0:0/14:0)	LysoPE(0:0/16:0)	LysoPE(0:0/18:2(9Z,12Z))	LysoPE(18:1(11Z)/0:0)	LysoPE(20:0/0:0)
430.3023	447.3323	219.1102	376.1363	356.2691	384.3370	412.3679	410.3522	424.3320	467.3004	454.3076	450.3340	418.2478	200.1048	342.2769	426.3706	481.3162	425.2536	453.2845	477.2843	479.3003	509.3478
36.91103604	0	489.1562336	42.02978154	413.9804507	91.64000225	426.5103591	89.59515334	58.95708038	390.8610653	19.52971038	307.6812321	10.97709028	26.95772187	0.026969825	0.132576282	390.9728013	66.13267889	1206.278561	302.7287204	263.7880507	98.26673884
28.68100343	0	266.6400991	34.55138224	378.5867376	55.13018116	504.168653	188.5292987	102.1495318	228.2633145	2.751599789	104.063834	0.882632572	12.10581483	3.699746541	2.753707878	697.4279038	187.5915093	1515.122591	2128.157435	1324.399175	283.1228239
47.93028664	10.62212996	468.4542328	50.97187739	388.2970981	64.85239034	2268.048568	134.2558032	97.06963568	115.6828579	8.36240459	213.396602	0.142588355	41.74087573	0.327766071	0	159.4594535	90.80758984	786.8412272	230.1689209	334.1822564	41.09107789
22.93125992	0	640.4941077	50.83488411	520.667569	66.64324244	673.2119424	374.5040339	130.0917114	42.363459	4.065113005	203.9937442	0.077897992	48.50764831	25.08343415	12.46332061	146.4066133	28.15893588	327.4153312	291.915649	354.7517494	24.98338348
102.7059937	58.46717786	574.8680902	70.80902756	708.9280542	88.26750199	5826.396875	728.2017134	340.0797549	96.79633911	15.66274572	207.3091185	0.018756905	50.93376405	90.18768036	7.26567823	100.9834952	41.69310265	351.3557043	119.6981565	122.2987048	2.060646571
237.2146868	0.726580553	278.1902979	93.45716646	723.8352212	108.0029012	6492.884615	73.21900288	79.16942736	215.8960638	0.945541848	438.9544549	0.989263463	36.02631627	0.015047086	0	76.6780528	124.6912125	815.9798707	576.8062709	278.80124	8.555069695
77.65487811	188.8462328	770.4956689	39.32893968	461.6013139	81.65572146	610.8446128	605.0320741	154.74837	142.4807243	6.144394842	421.6754013	0	63.42934748	32.54942842	2.167558559	174.7157335	71.34975665	368.8669863	301.4571835	290.3996474	51.36801318
248.5962669	0.091905975	170.8111729	25.38083173	603.1800707	54.26503751	4124.765096	302.2782652	52.06062475	121.0442947	1.803953878	326.3269225	0.689856515	22.80925107	1.217991411	0.468523763	508.4094746	75.55998764	914.4062918	893.8652579	353.2568999	116.4133535
150.2611019	88.68841493	528.779536	57.57575256	390.0326256	85.18023808	10548.28281	107.6574148	96.76227048	48.01040844	12.15581345	260.6218134	0.113666889	70.37770381	12.77229606	1.313869436	96.41984594	64.9604781	328.0356146	204.2718886	210.1319923	9.769198069
557.743134	28.69950293	354.6056264	50.66651988	491.2540948	217.571798	962.9738069	101.2955314	74.39174743	27.29109928	2.347020376	365.0319925	0.535697012	44.35536246	0.039882829	0.712079388	232.5658084	18.73450977	516.2493059	1079.857807	698.1375252	66.61261205
104.6938937	84.89652387	762.6763091	123.8841967	452.7791018	81.08862152	1910.906741	263.0986915	90.71894312	218.8664581	284.8009454	329.6999104	0.098318193	127.3486227	58.39441196	10.80404869	393.6745497	163.1282717	597.8293914	407.8377204	818.0240994	93.29343411
115.279791	0	790.0708738	96.33609221	1131.028045	96.14975392	5834.239223	433.6836685	107.4093519	37.74387516	138.8142362	171.4137765	1.178479454	58.19525357	165.9071367	8.29883445	72.52107618	39.5683417	357.2473404	135.626271	153.178014	0.766112983
35.02751424	0	590.2709741	100.2031318	1446.523307	101.3970323	10745.18784	279.8225037	340.8260482	79.79128575	4.465205828	613.5369388	0.778723628	52.47091409	25.08275559	7.668237072	103.8528624	44.67240557	396.5851419	155.7328484	177.2051466	6.830879534
49.60301369	4.492567219	548.8487106	62.84164709	401.2219633	76.35535711	1843.587206	457.1315258	93.39944762	41.29537632	0.649772428	277.7470486	6.475423658	28.5464288	28.37296176	7.512382412	143.8615532	31.16567417	330.4722584	185.6672203	293.4466423	56.01005807
43.33667276	5.159261717	422.6714142	55.21664144	421.9561415	101.1198615	6105.179551	352.4957281	78.62746572	31.8577081	1.956189472	181.2550779	6.503065723	27.43797751	12.7520268	1.513486757	113.3224882	36.75842098	409.169076	572.0502024	366.4861572	33.92636585
19.57671395	0	214.3505694	51.99336161	445.3770554	105.9532182	1474.72319	168.3542703	166.7608148	74.55190939	1.596295906	155.9311778	29.12376152	36.48565625	1.153362009	480.1169579	718.2311623	128.9094219	724.0389968	434.3337897	490.2291147	184.964654
47.43713617	0.467952264	147.656187	31.82968032	78.08549573	45.70708658	970.193835	76.06656034	53.15012885	223.9179345	9.678467125	100.5750717	75.83704994	38.77531193	0.014507772	0.211554333	270.7734429	168.6794063	963.937617	481.2212011	435.9408075	109.5359265
57.3271188	79.38800737	134.6204864	13.10697169	124.8972157	57.73566265	349.0614651	25.42885851	34.39912879	140.0929019	0.157868566	138.6842612	336.0044364	25.26091584	0.017478454	0	934.5637028	161.467415	1237.582992	1407.517767	387.756177	883.8593119
154.0710256	0	111.4624906	34.3872101	98.71527431	17.12052745	45.52015526	22.13171513	41.85241349	95.06132014	1.403626124	253.5674703	19.03897749	25.42360051	0	0	112.3944596	175.2330401	707.0510452	828.8268409	274.9237241	57.48097434
106.4098538	0.405984063	265.6305848	40.22607093	86.23569927	72.70840864	154.5089969	102.2304135	54.29803699	152.4551125	3.507495212	251.7181497	86.0257159	14.15162537	0	0	105.6561078	113.3722734	592.7674952	1026.103071	410.1484106	40.40228126
27.4688214	0	162.0524319	24.01704046	181.2240518	27.32465972	544.670433	13.24925196	43.02144751	43.72401786	0.570244643	90.78741977	31.06057424	2.768925581	0.054942998	0.017399425	168.8618428	13.58834001	595.8671955	1636.37128	570.3180299	83.39052299
27.31833307	0.125118931	467.6306695	28.08510925	109.9217248	40.95748008	216.5032041	108.5340523	81.7231061	304.6512586	4.702975399	228.4805793	51.52572633	71.86318933	1.213553245	184.7790346	708.1681595	109.1797989	1853.227486	1221.608471	522.5315881	376.0726021
95.5314259	0.184374133	536.5331312	78.80370881	356.0972319	81.73054588	978.5063521	347.3484567	70.91230797	388.1655777	1.893133164	302.0815456	76.02950592	75.53098065	5.900843489	493.3270759	581.2674626	144.5652149	1230.300156	1165.709354	656.800876	117.0535786
61.55596523	0.107607836	466.383922	52.36184994	404.5299997	38.10751091	4862.61717	233.5018042	87.13490143	633.975348	6.417022597	314.038342	15.85895811	48.09460421	41.3077925	2445.399004	1449.407051	69.7465762	1430.65403	998.9879827	709.0691799	185.4132096
22.56318715	4.481981066	263.2005002	52.89948892	442.4489706	64.74939512	1730.506571	282.4660446	93.83309449	80.44098707	5.743224067	160.2779576	33.98148276	44.48486076	37.70233763	1257.094315	309.4541531	34.28738278	552.3061159	512.7737398	361.2396697	23.51579583
66.76173536	0.043515504	236.704907	18.96074096	542.6079202	66.91433706	3007.176435	66.80324249	34.26440152	169.2148022	6.062939132	401.8671072	299.014072	25.45467335	0.042818206	1.454645286	214.4558788	156.2724582	1017.812023	664.0693883	317.3630783	55.95329303
49.35628393	36.38997764	145.915696	18.1083235	193.5134478	62.91833226	493.874855	56.88714957	28.88750204	425.8866155	2.993488863	86.25206659	621.1227647	22.91555805	0.164869313	0.056654727	916.8303099	175.5045962	1375.380435	2718.955292	1195.722072	388.8019818
36.59214211	1.433109359	311.0156439	50.35485341	68.33225162	36.5132398	376.3508434	65.62404209	74.70306322	53.92476114	4.719753963	196.8892137	9.135152071	25.08149956	0.294788441	19.89835928	132.8695611	101.63423	1288.225772	4315.546283	1186.473488	13.77164344
29.3225978	4.725259287	109.5741909	25.36927385	99.15311288	43.44332483	476.0833487	320.1858317	94.34110604	281.2253911	3.5980427	121.6755166	242.8289756	19.43404308	0	0	755.4031803	486.3708212	2090.201939	3660.247503	1089.064624	264.0812927
35.01743147	45.50970814	201.7963434	36.10842232	148.1842849	34.92614248	678.8313171	48.30506597	62.75614403	1375.954395	5.112339457	279.0080597	60.74924871	62.5497369	0.038828434	0.045918895	1726.530053	572.7428148	2532.383557	1920.430385	971.4033373	1069.592039

99.4048366	3.375053083	786.5867107	79.38682937	599.3656631	76.9071676	347.0530952	482.7279885	83.34961459	997.3639843	3.048609501	409.5826467	31.51597598	57.46955396	33.88166816	2178.075089	405.5833836	300.8763653	1588.14554	292.3540817	311.2441665	3.967439953
53.46069674	0	490.1107671	44.05546411	1115.100195	48.66101208	5457.294389	445.4324013	69.88565548	166.0985847	5.887579952	162.7581694	79.75903203	41.62891135	1.66040648	763.4218049	372.4254207	160.6327178	1150.810274	1436.921441	479.5681046	105.9632468
53.17548995	36.87076342	694.6139265	62.78827407	753.0774095	135.055194	3298.251073	594.5341995	93.14898486	97.6292873	6.217569817	518.4318016	20.49687367	58.56137499	3.852358119	537.2112475	209.9286061	114.8106626	641.2177958	334.9721584	271.160338	62.80567686
213.8927183	0.081047685	370.6770425	71.5947605	521.1317626	125.6830579	5487.090769	132.1544421	138.2884486	37.52892576	39.33630627	462.2580602	2.478646029	28.91321818	123.6194399	2619.748315	47.11308381	57.45240962	141.7327662	96.56647885	120.6785363	3.661915725
195.0560941	0.463838317	980.4002911	71.17032391	470.760325	79.63116606	612.1894282	127.3459369	201.785133	565.900112	0	262.8531646	110.9427317	38.74618207	5.295560905	128.5160659	698.8759241	102.3025761	816.3175992	1876.223248	720.8654393	29.2702549
33.10080303	0	60.37865387	25.91253948	374.3742175	77.17618142	535.645692	114.2975877	62.08984273	190.9831573	0.092980501	163.7178555	17.4473755	16.36019296	0.144870534	14.62339685	942.7205717	215.3306183	1467.942224	4029.362096	1676.474181	297.7252094
70.93511078	0	287.5220574	50.73540206	367.8830862	78.22792348	1364.043468	262.1893544	84.98203536	208.79124	0.628872298	225.7416739	38.93016415	29.7182316	1.981532242	1182.145035	450.1924318	110.0653485	1045.891461	595.914002	370.8665015	99.64148831
76.54636106	0.039539437	114.7715867	16.5800231	317.1161434	36.31359923	1640.619553	47.93180033	50.43531368	210.2412815	0.230707531	55.54149415	398.5907481	13.8090923	0.021353984	1.405492712	260.1330943	93.90318734	1101.817008	11266.65727	2583.722667	128.3178226
73.72391575	0	799.4605942	81.75088048	306.115139	64.06563181	472.6176075	339.7810048	143.5737589	220.3118401	9.952831752	332.4842657	30.58998232	32.3588369	7.400812933	980.6984297	370.7947917	56.71867293	624.9220809	385.9521688	350.5654261	33.79092058
10.95110316	0.308359292	49.98536294	29.33106532	622.0483154	44.18754808	1448.236861	89.95097566	77.27305974	98.03731156	1.299892142	115.3533997	299.9331555	16.27506169	0.028707154	0.873632357	494.3215615	83.98025137	722.6764412	8435.314286	1568.097819	267.5147969
27.83532418	0	400.5424856	89.27223818	106.8406634	35.25216605	694.9587487	113.5832701	68.46605708	83.53548438	2.8566108	253.4632684	92.21513167	40.4141744	14.43416628	1924.362076	142.4840807	43.91820009	582.7159892	422.730785	274.6964306	35.00313234
25.92558181	0	176.7806913	25.58978788	215.5687937	113.1027562	547.0555618	65.13846699	45.97717376	219.8032457	1.165129215	105.583528	0.013268272	3.418807954	0.032168959	0	187.7454006	251.655876	844.0261402	1173.697438	417.8590811	72.82979829
42.90768972	0.040543565	164.5192364	37.14399234	677.2444258	89.05255051	448.9538701	353.9097029	97.2211595	568.8813142	6.598442855	159.9608572	0.058096806	12.48140137	0.211510934	0	762.8289411	227.0131004	1915.547231	3301.948803	1579.440569	350.6244557
81.69141422	2.434775837	665.2198051	78.22813957	113.8272427	80.70721206	2286.328715	85.53901737	167.1754514	154.7981797	6.382500106	439.0368614	0.135403206	25.1459185	48.66210637	0.818534783	149.4599927	416.9363862	762.8779494	289.5525963	348.4788569	28.36992935
7.144327434	0	82.94374166	21.32921833	490.0036987	26.04193722	241.0371513	46.86126596	66.34462438	837.1952455	0.188176183	75.58125233	0.056478755	5.250782374	0.048016823	15.46885357	1673.801931	254.3268834	1766.91385	5948.506966	2038.268479	660.9566757
35.92845863	0.087789884	253.1602427	32.6995232	232.3225311	134.6557188	230.5490506	38.1112759	94.72731415	95.11378791	2.483053937	164.937695	0	10.65063671	6.987337592	0.018798849	1945.335581	197.0491465	1628.050332	351.6184531	303.2647428	418.4246154
64.37438298	0.819416003	153.7134218	54.69723116	168.0577447	24.31775055	122.5860742	46.07226181	50.21371517	97.76298741	1.069530857	79.46273649	0.009868811	3.437554744	0.34791485	0	74.69547555	60.26417465	765.8113391	297.9143551	119.2725744	5.252138842
19.97469475	0	168.3182396	85.86645622	188.9595363	62.97520731	425.0753077	86.07477223	54.90044702	1069.74535	1.818368039	166.0181559	0.072911931	53.90918493	1.523529164	0	516.4011252	309.2413651	1804.713263	1651.581753	593.643748	69.78998905
10.02806924	10.65386922	142.825828	29.7413822	483.8561833	38.63139304	1164.982646	239.9253207	63.76735423	287.0829183	1.021997817	115.4748685	6.41787393	22.69256976	1.242843431	233.7959822	1770.855764	212.788776	2695.70978	817.9712823	1541.039073	634.8900741

MG(0:0/18:3(6Z,9Z,12Z)/0:0)	MG(0:0/22:5(4Z,7Z,10Z,13Z,16Z)/0:0)	Palmitic acid	Pantothenic acid	PG(10:0/10:0)	PG(18:0/0:0)	PS(15:0/0:0)	TG(12:0/12:0/12:0)
352.2613	404.2918	256.2401	219.1105	554.3209	512.3164	483.2589	638.5478
75.28743846	202.0033602	174.2772241	3265.812573	99.03756907	372.4309339	101.8371424	1024.530601
25.23394057	48.74679582	179.6515992	722.4352538	134.8073562	169.9249639	134.3402424	50.32979299
47.1196916	393.6762726	121.744606	1698.000262	117.6938682	395.6402582	18.57241247	167.2784403
27.92551933	104.217363	42.68871662	1917.380721	669.2371412	187.8767257	23.48041089	2.874571765
32.8440976	2724.713509	63.31229084	1057.39831	325.6837591	2153.770829	0.167396055	55.21544526
36.8113909	276.8670919	118.4909369	940.2893617	119.9166692	421.266145	16.21401259	43.86898196
61.29875774	655.9799724	73.41628654	2989.16787	199.2173478	1013.092041	26.96937862	112.6321848
24.75003744	336.8756363	159.222166	1188.897757	93.71954147	860.5590228	77.13315385	4.534072184
18.3457449	333.8832892	47.54981645	1612.419924	141.1615052	200.7359586	2.131381541	49.85147607
79.64411828	687.5174674	62.72359474	2022.777779	276.2758307	814.3507003	0.573522267	36.15166205
76.42070009	244.0243986	101.4131332	3447.597188	1173.269279	597.9104398	24.17993153	509.2408452
144.1891823	493.6092876	55.0929549	2261.223982	494.7105976	1071.390644	3.363692754	545.3550865
19.61676338	892.5344189	94.953045	2110.314554	478.7296015	841.9438589	10.16762105	20.86221741
134.4579868	446.9433982	65.37912643	2394.738993	285.7968797	681.1440856	5.105079462	253.8034649
240.7538027	229.7016133	61.96224088	1677.523363	341.684799	733.1590349	24.42815107	625.591726
54.94680105	365.8839587	113.0528803	623.4092504	128.7485092	1.922002462	76.37781608	28.58625237
26.31238327	43.82677475	173.4648179	728.1993778	29.84561787	3.306817583	70.43954798	291.1283612
10.16375902	30.59916306	167.9556507	1693.121706	108.0731989	18.52150489	87.50281214	109.1881564
15.95447239	54.70757641	67.1064566	466.2876045	67.66289129	92.14354825	33.88607152	7.736389145
34.66457014	294.438073	73.65698106	1977.772721	18.81418944	363.2741759	84.58237272	35.05702546
3.489419134	29.61761335	62.42593585	770.837274	14.99398722	248.8197198	427.089689	2.20128513
51.41710365	900.5626036	193.0173356	2262.785285	55.35143416	1041.691495	108.2260263	50.34913466
59.00777482	729.9264979	127.2528624	3039.712613	190.5434699	91.65028659	195.4612851	264.6350645
27.60103724	171.5004573	168.643829	2379.624527	120.5753148	24.70119792	137.9005566	14.21805497
27.42783605	299.1896159	81.47603958	1143.270349	99.24369477	32.09055567	19.87173771	397.7170934
78.51288989	573.1733505	135.0361247	1651.015875	65.31595996	13.80444181	73.80303275	479.7625119
39.84124926	45.45305453	165.178168	1314.463003	20.49075567	71.38770325	124.9574084	251.6183427
19.04906237	35.2977044	39.44958544	1451.789884	47.4923035	16.93515322	53.26605316	27.74998721
27.52545762	32.07764055	244.86274	408.4252925	19.91366814	14.05950131	310.5612236	7.763730162
12.53646001	152.0981486	349.9901682	1808.712035	29.53718869	22.72504713	347.8920514	83.13003016

106.2663252	216.5920394	166.5167137	2333.412706	61.93628364	32.41137986	155.5881834	328.9877579
21.4977389	103.4616845	151.7465272	2095.286476	217.5971332	14.07642535	114.230498	8.28814333
55.46383122	538.5706797	90.22694961	2172.640035	293.5364185	164.5739827	72.76155333	36.93765153
61.4780325	570.9347682	33.63415014	1358.492731	157.462379	69.91425433	9.868399083	270.2411847
14.77392459	216.4143034	174.7360412	2300.053426	140.2668167	6.296936789	46.62805312	0
26.77775146	106.6856794	197.5708116	185.0792308	194.7488548	9.571568813	97.12572067	69.52643309
27.51830561	133.2780032	157.0139033	1314.68667	49.88891162	8.065862666	72.08588583	30.22068586
5.578000913	2.372150179	121.664845	560.7890319	48.69168924	0.800721161	289.9347655	16.62919804
35.81040358	530.8582499	106.2882249	2446.811407	58.34746676	6.12021337	5.770970572	57.59802764
14.17364308	147.2787996	156.674467	174.3359508	75.3306178	3.616970461	379.2815416	10.24672117
28.55390575	121.0510238	84.75202192	2456.448517	61.01110761	9.574819736	88.99885307	62.71662535
10.87000911	38.0747243	126.179626	605.0416699	0.217735242	1866.63703	257.6427433	43.86693254
38.16759182	386.504603	306.7774242	376.1147547	7.182168041	1112.336938	316.8366876	226.9165252
39.11054776	606.347615	65.53588662	2406.157831	57.02481465	639.6779201	17.53521705	259.415585
10.26712468	81.84904515	360.8512354	311.1015775	53.75341801	344.1428623	244.2568061	3.406961143
5.254942876	163.004753	50.74103927	1053.423226	32.60677465	1332.907651	256.8368734	63.28684598
6.514707362	10.64151286	136.6236532	784.4840968	8.307553366	262.3848792	34.90746768	17.97259059
85.76349507	103.1121026	249.4799526	1184.541407	10.94175865	385.8547564	346.8429271	57.44662864
112.4991629	68.76250379	270.4289445	681.0859337	321.3281786	547.9687418	309.3725566	21.02367399
