# Supplementary material for: Dysbiotic gut microbes may contribute to hypertension by limiting vitamin D production
Source: Clin Cardiol. 2019 May 28;42(8):710–9. doi: 10.1002/clc.23195 (PMC6672427; doi:10.1002/clc.23195)
Supplement: Supplementary file 3 — Table S2. Absolute abundance of 20 genera discriminative in HTN. [file CLC-42-710-s003.pdf]

Sample	Faecalibacterium	Roseburia	Akkermansia	Clostridium	Oscillibacter	Subdoligranulum	Ruminiclostridium	Butyricicoccus	Anaerotruncus	Butyrivibrio	Intestinimonas
CTR1	564264.2647	161480.8755	585.8640965	385021.4679	404211.2113	379712.1137	25214.50262	10306.66255	14753.18755	3102.42157	15029.06619
CTR2	2902024.654	132279.8925	189406.0629	564302.8383	762915.4001	119868.1925	23230.38666	8836.498826	21106.36209	9934.918145	15569.64369
CTR3	1621100.736	240822.1216	1022.230169	434065.3507	1175715.287	379543.9469	34747.14563	15440.76198	48057.57764	19602.46413	24077.55293
CTR4	2366179.131	439257.0339	567.0516411	382320.6232	381826.5664	79279.389	55579.25067	7308.200421	15225.8675	24041.54128	6833.372866
CTR5	2778849.838	221735.0024	608123.1163	1246520.047	718522.3971	605975.0483	51562.92747	18459.42325	65529.26612	26543.22776	108997.7637
CTR6	4741893.951	604305.202	9979.185626	1454179.709	788037.4714	581842.6373	50926.92183	27998.01567	37296.80939	32406.54992	18962.24849
CTR7	1613677.768	1135129.581	105.2069278	806989.0161	718370.8172	107299.2493	18006.6815	11378.8278	15744.60255	15828.82166	12622.79661
CTR8	603866.2653	124149.5034	14.54155094	256170.894	10056.02256	9474.38745	2087.557378	1428.828029	4464.392777	2848.891406	2409.603438
CTR9	1106947.03	233895.5789	1016.662603	410219.7107	107146.4549	70631.04777	8540.14712	15470.04283	15714.09665	6957.344386	4071.011305
CTR10	2385198.761	3628094.596	2814.371196	441201.5247	32597.85621	463616.7431	27399.06945	7863.853303	13370.72769	26769.95562	53117.34931
CTR11	1716149.71	712568.7465	1966.26986	484067.1603	241884.0892	204589.9536	111320.6856	5311.615275	16789.0623	24433.0193	10183.04342
CTR12	2375574.095	986422.9507	98.27883406	1153749.019	72098.1885	19091.07074	20486.05965	23053.35522	16129.6982	10290.56689	4801.231248
CTR13	3260077.011	141658.9652	220164.1446	335619.7842	1341833.209	701671.4209	52101.83268	40549.47967	35727.94201	17160.61527	43156.71412
CTR14	10990.05409	724279.2557	1497751.664	560997.1029	52208.64581	8810.555063	14680.75156	23156.25254	15711.76829	7122.589476	8861.469688
CTR15	435735.8404	619539.3991	222.5443846	938722.796	297382.6629	31405.06869	13036.46784	35753.88601	8638.867876	17794.82231	4995.064327
HTN1	4130408.979	725541.4375	87.7812659	486688.9213	1460767.328	481952.802	44087.14659	33456.2529	30425.81661	16905.45587	26166.89878
HTN2	884612.0318	55473.41127	8.160464748	243938.3043	24800.79559	3329.285228	4135.008732	5222.491595	2453.984768	2551.753692	4477.362919
HTN3	510502.5437	98173.5037	3.085929513	341492.9934	15145.8797	1787.298425	2505.512645	2142.098254	2873.92768	5729.772642	902.3641909
HTN4	4043205.783	59186.72887	67.26466793	88539.9749	140772.2856	34925.45038	6413.058527	5904.811222	3939.610328	4623.438605	4596.444559
HTN5	932546.0811	406140.828	2977.849183	349713.2814	117419.135	180866.4972	15995.85971	19154.1582	4611.278746	16307.63932	4690.471492
HTN6	52261.74047	233182.2543	96.67673514	500129.4973	4739.055023	3705.996615	3052.330235	1514.208048	2617.146124	7262.100932	997.3333429
HTN7	1968221.816	216969.619	20095.73136	1682491.329	2131262.169	215695.0455	113209.6251	30138.22108	262657.228	37180.0847	43245.91534
HTN8	551285.6786	162726.6341	3818.651106	382036.0967	13807.27921	4402.30007	4860.188	3002.120273	2702.068465	11074.91363	1212.788999
HTN9	17314.75873	15617.16008	9.274701669	581956.1377	16071.80739	2812.205465	2704.104631	11816.45518	6021.014089	2070.253849	1487.764223
HTN10	107335.6432	19921.65355	839.3674224	100534.9654	78808.34083	13807.8709	3416.249896	1208.916978	2477.069717	2825.980014	1107.67958
HTN11	345225.0036	108061.9044	2724.645659	134996.302	7357.548292	11364.16363	1258.727853	3300.950178	2211.178582	5457.194707	1021.940017
HTN12	2118345.814	151766.3382	9.23959151	422109.8998	87877.5138	76985.76922	7714.501876	10235.85913	12827.70298	3728.562457	5794.924556
HTN13	15509.65816	14089.36467	1.06840639	333572.8141	8305.032625	3513.525982	3446.004218	4327.399706	4191.571562	4242.447746	348.3030594
HTN14	2065692.002	138777.056	202.682975	177072.7302	439423.0467	307808.3325	15836.39832	14946.59408	14418.16214	7366.482053	6406.41976
HTN15	3925.661466	84579.7098	2.63763296	204398.0812	11290.20464	1142.070645	4533.60142	5213.66959	1660.540556	1164.296298	906.8942104
HTN16	6013252.959	2115889.207	57.47527832	2875168.272	50457.13846	19803.21523	10384.66992	42527.8732	7900.098553	21397.09952	1773.615812

HTN17	1167808.146	1217282.41	29.76527125	1045266.683	69635.32653	165969.2537	18176.99224	17288.8871	9520.674772	32613.63738	2799.126209
HTN18	3952.129007	1828.728099	28.2428444	18254.67753	972.3929823	42746.71789	329.7731848	313.3649376	196.0955342	196.4519598	126.1943341
HTN19	445771.5501	195911.7648	2389.718026	343406.4678	101959.1205	61501.75278	12963.46497	3833.01332	13508.14507	13977.1817	3498.159801
HTN20	6818509.847	378544.394	295.5249053	297241.1569	152820.7137	142403.5155	15940.68883	12478.04541	20716.60235	9241.991242	7522.993033
HTN21	1684505.711	93296.1991	26774.63269	283971.6363	328252.0455	190002.0145	10617.694	4045.824214	8964.469407	11235.44572	5418.057298
HTN22	7599603.534	861977.8622	79.19520989	490742.7126	154502.7726	148446.9368	20883.27778	12289.15157	16523.72406	8755.074401	13204.92982
HTN23	4023520.481	722549.1467	14.02887541	694864.3069	50260.34492	44587.93192	14190.94407	5729.918169	17442.13439	13898.3163	5274.223557
HTN24	45616.47061	15202.52192	73.08581551	121395.832	45838.06969	117121.4213	7929.869423	72956.41565	8455.673339	379.2244921	2204.448555
HTN25	133409.3885	8816.765151	773.4520565	188166.7137	76833.34499	18156.12239	5932.681212	3632.14375	6189.886585	2587.818141	2207.241121
HTN26	199432.2969	460998.7866	7.970828415	882202.3501	12609.69436	5076.393617	10047.07863	2784.664721	5536.636752	21271.06349	1155.180023
HTN27	2373.069595	6413.330135	7.020442765	298601.1415	10692.64051	4302.47547	4660.295091	8550.075985	1902.504989	5066.699295	334.1112093
HTN28	7474299.529	61897.01954	128.0602125	376723.9065	856371.7273	208773.78	22966.99205	37046.35467	22560.64815	9641.579116	17372.48864
HTN29	1336886.028	729373.9116	85739.65798	1802945.533	1104851.316	308212.2298	86990.66898	14071.38461	61618.81159	33915.7674	50758.5917
HTN30	7430917.177	106156.5562	662.5564667	321312.8799	480658.2077	116505.3081	24849.3117	8796.970718	22655.88958	11137.47152	11730.48555
HTN31	1909995.704	2210481.535	153.1815102	575039.1108	67901.6098	70624.87969	27770.86721	3999.882973	9007.253935	81028.58547	2720.311688
HTN32	1845693.245	375016.1225	123.0132055	476377.157	166538.3057	152309.3066	40380.96929	20723.36688	11000.30565	12940.34758	6542.45979
HTN33	3890113.529	605260.9202	52.85777424	429261.6655	1741797.21	238908.5971	24718.17061	12991.63936	26040.19961	16295.80565	46639.49881
HTN34	525130.2983	4082529.386	76.19861042	698911.289	41784.58219	65285.87656	14414.31513	10820.99729	13223.33886	41419.56543	2088.877436

Pseudoflavonifractor	Paenibacillus	Pyramidobacter	Holdemania	Marvinbryantia	Oribacterium	Treponema	Robinsoniella	Acidiphilium
19970.64762	8604.601487	1428.826101	15352.66706	4112.886639	6259.574615	3311.133839	2116.018304	784.9262674
12514.19572	5226.837646	5077.109164	15156.80905	3104.480328	4729.48293	5260.617401	1791.470543	698.9769885
25870.10283	20281.61042	7333.047716	12461.15418	7751.303188	5934.139238	7031.946338	12941.11412	5087.084768
8334.614883	9116.302104	1109.196875	7263.324277	7604.335384	5505.634633	5343.484151	5901.364469	2703.100992
65871.37137	20662.738	3871.219409	23865.39678	14285.92961	6721.428182	6541.278739	6018.112919	1818.714131
19613.90948	20360.00341	4294.58255	9766.882828	27680.9687	10210.89579	6756.62236	11824.48266	1276.209707
10804.06212	6316.739271	3364.021726	14891.16231	4591.38722	5244.171027	5736.032813	4855.857582	232.6664529
696.9636388	23704.71748	189.4742389	2603.832105	2006.916677	4272.974	2661.85961	335.2037985	1.941893177
4001.101949	2946.116302	1772.183716	2666.447025	2787.194993	4272.241408	1921.423756	1488.995286	105.324044
19195.14604	7658.945578	455.6734705	2680.904403	9226.898171	5722.803623	1813.01927	6074.716797	506.7252983
9173.532199	12744.11555	1634.240566	5286.474774	8466.886308	5784.144592	5069.41723	5014.527427	3521.419832
8227.912396	1975.654419	333.3522307	7322.90898	6662.220513	9366.912218	3376.715959	2002.65078	336.2459309
23285.83265	13425.3693	3285.336854	12811.19383	10141.80119	9630.665166	7090.034039	6552.849093	936.726743
20095.09237	5492.127103	260688.4838	1405.641984	7738.357566	2612.999026	2707.777345	7126.279397	0
6956.841085	3688.906399	1226.11137	4298.091218	3444.932022	4618.098915	2260.692794	2405.825452	16.99550831
29213.323	9623.217009	5164.215605	22689.04474	9652.695734	9276.823327	6401.528859	4016.416324	342.0574469
8259.894916	926.1851475	141.409481	495.1789278	1078.253773	1222.188292	804.0858224	219.8136293	3.364622388
2776.716834	2133.186038	7.799110114	450.4948063	1854.883407	1980.822921	1867.33132	761.5594108	28.90943162
2928.661941	4266.428052	820.2823902	1593.594653	3829.061478	4353.235365	2878.840717	1962.961303	39.30665013
4575.666172	5233.773063	1273.29072	6124.552998	4259.37894	4162.702329	3700.664977	2973.872577	144.6843003
2573.068399	1283.138809	192.6234545	2161.06358	1576.35163	2286.514252	4507.462874	2253.127477	93.43696596
62081.90293	52225.12225	10318.65674	22416.97994	9888.835614	9636.257149	18389.11022	13119.05285	7746.419935
1482.200458	3234.874203	90.11874648	417.4311219	2073.864203	3940.714152	1248.557133	937.1319031	56.22335536
2381.351494	1192.754728	78.0804395	1071.780095	1795.509858	5202.086777	435.4041592	400.9090169	5.382031807
1310.989944	1493.82076	160.1155845	598.2278176	562.7698657	950.7194038	5514.46938	455.3205082	21.97496737
1735.921088	854.7953366	309.6159103	319.4552588	1353.042578	1398.614738	4270.299456	510.4077494	2.232798894
4620.328809	1324.221712	387.167504	2060.628704	2921.332649	3497.48773	2669.427	854.619938	25.71807082
4249.442253	2098.879749	33.86905671	2020.730231	3277.116867	3070.19805	1479.323888	1491.949689	1.581241457
9052.898368	19987.69553	1517.759281	4086.478857	2670.348353	3187.485988	2444.806157	2005.942581	295.867357
1108.260288	1515.923737	208.616487	780.466349	498.2776465	436.9934588	371.2328587	478.7734909	7.644315751
17315.53215	5729.382044	7.749432788	9703.205204	5334.864884	5754.186272	3978.413542	3323.474932	207.3126602

7815.334821	8465.916519	733.9875952	3602.404646	3882.183049	7356.408526	4413.043828	5526.513242	128.4363744
285.8809993	1185.568626	5.548098198	55.00314109	81.29380703	91.85446264	228.1840068	123.9746835	13.48217725
2993.148614	3264.961066	228.2953182	2699.218388	2228.254149	3751.269454	1623.050124	1730.820817	177.6222664
8050.142649	2823.291985	725.5034684	9484.463225	5379.841212	4224.719262	2798.001969	3944.01108	132.7252828
7005.738719	3230.323056	15457.13558	2455.515825	2432.248257	2534.552059	2161.051755	1877.825041	56.72335772
14944.57174	4582.881893	2705.593827	7244.877648	8679.335988	6540.357384	5271.821013	1992.273536	56.81121219
6680.623492	3168.225472	647.6950688	16031.98081	5250.790055	12463.35384	3341.557823	3072.704001	83.84101911
5566.159117	3238.291818	6258.074924	2627.921908	2476.003597	1012.47732	2144.858996	730.6206632	17.96322506
4165.441117	2634.143603	443.8485765	1917.932071	4356.615456	859.6050459	906.0916686	543.7716288	107.6122835
4021.985521	4568.729461	368.3441736	1688.687091	3597.035918	6300.078618	3177.799748	2626.768556	61.11471217
908.9696958	2040.37787	91.06780763	595.319972	731.3673261	3217.40686	1455.902198	302.4533281	0
14337.4899	7959.066562	2441.39194	15779.65684	3185.96714	3557.793666	3533.292306	2438.437862	747.3287169
35216.74487	25661.5274	5345.086651	15302.68676	13112.22434	9766.410514	13110.14468	6347.811678	6153.971797
12460.39782	9219.501182	3584.130433	12298.24233	5519.456715	5702.712173	6073.469173	2270.208473	16354.6682
2467.442425	13258.25603	1059.871275	6464.444534	16170.04811	6613.828829	8833.327818	5328.658163	332.7500875
6444.450558	6150.669128	9783.669884	2237.139064	6049.227792	4012.003019	3317.173379	3414.18751	256.6847672
21701.60685	6568.727361	2517.874296	12672.07375	6050.525715	6457.938323	5826.912758	3626.938034	219.091753
5065.039869	5557.071355	692.7905938	2053.684136	6339.240085	3848.098302	7450.502613	3993.778558	17.59404661
